# A comprehensive atlas of full-length *Arabidopsis* eccDNA populations identifies their genomic origins and epigenetic regulation

**DOI:** 10.1371/journal.pbio.3003275

**Published:** 2025-07-15

**Authors:** Syed Shan-e-Ali Zaidi, Sara Shakir, Hanne De Kort, Devang Mehta, Vu Nguyen, Ruben Gutzat, Hervé Vanderschuren

**Affiliations:** 1 Plant Genetics and Rhizospheric Processes Laboratory, TERRA Teaching and Research Center, Gembloux Agro-Bio Tech, University of Liège, Gembloux, Belgium; 2 UMR Biologie du Fruit et Pathologie, INRAE, Université de Bordeaux, Villenave d’Ornon, France; 3 Laboratory of Tropical Crop Improvement, Division of Crop Biotechnics, Biosystems Department, KU Leuven, Leuven, Belgium; 4 KU Leuven Plant Institute (LPI), Leuven, Belgium; 5 Division of Ecology, Evolution and Biodiversity Conservation, Biology Department, KU Leuven, Leuven, Belgium; 6 Leuven One Health, KU Leuven, Huis Bethlehem, Leuven, Belgium; 7 Leuven Institute for Single Cell Omics, KU Leuven, Leuven, Belgium; 8 Laboratory of Experimental Plant Systems Biology, Department of Biosystems, KU Leuven, Leuven, Belgium; 9 Gregor Mendel Institute of Molecular Plant Biology, Austrian Academy of Sciences, Vienna Biocenter (VBC), Vienna, Austria; Sainsbury Laboratory, UNITED KINGDOM OF GREAT BRITAIN AND NORTHERN IRELAND

## Abstract

Extrachromosomal circular DNA (eccDNA) has been described in several eukaryotic species and has been shown to impact phenomena as diverse as cancer and herbicide tolerance. EccDNA is thought to arise mainly through transposable element (TE) mobilization. Because studies based on short-read sequencing cannot efficiently identify full-length eccDNA forms generated from TEs, we employed the CIDER-Seq pipeline based on long-read sequencing, to obtain full-length eccDNAs from *Arabidopsis*. The generated eccDNA datasets identified centromeric/pericentromeric regions as hotspots of eccDNAs with several eccDNA molecules originating from Helitron and LTR TEs. To investigate the role of epigenetic marks on TE-derived eccDNA biogenesis, we studied *Arabidopsis* methylation mutants *dcl3*, *rdr6*, *ros1*, and *ddm1*. Contrasting the TE-suppression previously reported in the hypermethylated *ros1* mutants, we identified activation of TEs in *ros1*, specifically of *LTR/Gypsy* TEs. An enrichment of *LTR/Copia* elements was identified in actively dividing calli and the shoot apical meristem (SAM). Uncharacterized “variable TEs” with high eccDNA and expression were identified in the SAM, including *ATCOPIA58*. Together, our study reveals the genomic origins of eccDNAs and delineates the link between epigenetic regulation, transposon mobilization, and eccDNA biogenesis.

## Abstract 2

Previous studies of extrachromosomal circular DNA (eccDNA) in plants are based on short-read sequencing and cannot efficiently identify their full-length, generated from transposable elements. This study uses long-read sequencing to obtain a comprehensive map of full-length eccDNAs from Arabidopsis and identifies their genomic origins and epigenetic regulation.

## Introduction

Extrachromosomal circular DNA (eccDNA) are found in eukaryotic cells in addition to chromosomal DNA and can be visualized microscopically by staining metaphase DNA [1]. EccDNAs represent a pool of circular DNA molecules that exist independently of chromosomes and are not associated with histones in the canonical chromatin structure. Evidence for the existence of eccDNAs in eukaryotes was produced more than half a century ago [2], initially in boar sperm, and later in plant systems such as wheat callus and tobacco leaves [3,4]. Despite their early discovery, the biological significance of eccDNAs remained largely unexplored, primarily due to the lack of efficient methods for their purification and high-resolution characterization. Only recent advances in sequencing technologies have made it possible to describe eccDNA structure and function [5]. Growing evidence reveals an unexpected prevalence of eccDNA in several organisms and in various tissue/cell types [6–8]. The generation of eccDNAs is not completely understood but there likely are multiple sources of origination such as rDNA clusters through homologous recombination or active transposable elements (TEs) through circularization of linear extrachromosomal forms [6,9]. TEs have been reported to produce circular DNA intermediates when transcriptionally activated upon stress [10–13]. Some of the potential roles of eccDNAs in biological functioning and evolution can be deducted from a well-studied eccDNA-making retrotransposon *COPIA78*/*ONSEN*, which was first found to be activated by heat stress in *Arabidopsis* [10]. This TE primarily targets euchromatin regions for TE insertion and can impact gene expression [14]. In addition to *ONSEN*, several other TEs have been reported to integrate preferentially near genes [15,16]. TE mobilization has also been linked to increased genome size with considerable transcriptomic novelty [17], and retrotransposition bursts have been linked to the complex reshuffling of parental sequences [18], indicating some potential functions of eccDNAs [19,20]. DNA methylation is one of the major mechanisms used by plants to control TE activity [20,21]. While current research has begun to uncover the relationships between eccDNA and key epigenetic factors [22], the extent of eccDNA involvement with other epigenetic mechanisms, such as DNA and histone modifications, remains an area requiring further exploration. Together, these studies indicate that the eccDNAs arise through the activation of TEs, but the functional roles of eccDNA remain understudied, particularly in the epigenetic context.

In recent years, using high-throughput sequencing techniques and bioinformatic pipelines, various methods have been developed to detect eccDNAs, such as mobilome-seq [12,19,23], circle_seq [24,25], ECCsplorer [26], ecc_finder [27], and ecc_caller [28], eccDNA_RCA_nanopore [29], and Circular DNA Enrichment sequencing (CIDER-Seq) [30]. The CIDER-Seq pipeline allows unbiased enrichment and highly accurate assembly-free identification of circular DNA molecules using long-read PacBio sequencing [31]. Since CIDER-Seq relies on the number of concatemers to tag the reads as circular (identified as the number of rounds in the deconcat algorithm), this eccDNA identification approach is reference-free [30]. Recently, CIDER-Seq analysis of glyphosate-sensitive and resistant Palmer Amaranth (*Amaranthus palmeri*) has revealed the presence of several diverse eccDNAs varying in size, repetitive content, coding sequence, and motifs associated with autonomous replication [32]. This is in addition to the 400 kb eccDNA previously identified, harboring 59 genes including EPSPS which confers resistance to glyphosate [33,34]. These studies indicate that the profiling of eccDNAs can reveal functional insights into unknown molecular mechanisms.

In the present study, we took advantage of the CIDER-Seq [30,31] to generate highly accurate eccDNA profiles of *Arabidopsis* tissues under varying conditions and genetic backgrounds. We hypothesized that genomic context can play a major role in the regulation of eccDNAs and therefore we analyzed eccDNA datasets in their genomic context. Taking advantage of the transcriptome and methylome data we characterized the role of epigenetic hallmarks in the regulation of eccDNA and identified potentially intact and transcriptionally active TEs in *Arabidopsis*.

## Materials and methods

### Plant material, growth conditions, and stress treatment

Prior to germination, *Arabidopsis thaliana* seeds were surface sterilized and stratified for 2 days at 4 °C. Before and during stress treatments plants were grown under controlled conditions in a Panasonic MLR-352-PE growth chamber on solid ½ MS medium (1% sucrose, 0.8% agar, pH 5.8) at 21 °C with 12/12 hr (day/night) light cycle. For the heat-stress-induction of *ONSEN* transposition, a prechilling step was performed to increase the relative activation [10]. Seedlings were first placed at 4° for 24 hr within the growth chamber. This chilling pretreatment was followed by 24 hr with the chamber set to 37° for the heat-stressed (HS) plants; control-stressed (CS) plants were moved to 21 °C. All *Arabidopsis* mutants used in this study *dcl3* [35], *rdr6* [36], and *ros1* [37], are in the Columbia (Col-0) background. Samples for *ddm1* were prepared as part of Fluorescence-activated nuclear sorting (FANS) experiment (described below). All plants were grown to obtain three biological replicates.

### Calli induction

Calli tissues were induced from *Arabidopsis* leaf explant as described before [38] with slight modification. *Arabidopsis* seeds were grown on ½ MS medium at 21 °C with 12/12 hr (day/night) light cycle for 3 weeks. Mature rosette leaves were harvested under sterile conditions and leaf strips of about 5 mm × 2 mm were cut with midvein going across the width. Similar size leaf strips were transferred to *Arabidopsis* callus induction medium (MS agar medium with 100 µl 1 mg/L 2,4-D and 100 µl 1 mg/L 6-BA). The plates were shifted back to the growth chamber at 21 °C with 12/12 hr (day/night) light cycle until sufficient callus induction. The calli tissues were harvested in liquid nitrogen for DNA extraction.

### Fluorescence-activated nuclear sorting (FANS)

FANS was performed at the Gregor Mendel Institute as previously described [39]. In short, 300 mg of above-ground seedlings (7 d.a.g. *ddm1−10* and Col-0, both with the pCLV3::H2BmCherry reporter [40] were collected into 3 mL of nuclei isolation buffer (NIB: 45 mM MgCl_2_, 30 mM sodium citrate, 20 mM MOPS, 0.1% triton, 0.5% beta-mercaptoethanol, protease inhibitor cocktail (Roche #6538282001) and RiboLock (Thermo scientific #EO0384)) on ice (3 replicates each). Nuclei were isolated using the TissueRuptor (Qiagen #990890) and 30 μm filters (Sysmex #04-0042-2316). After centrifugation (1500 × *g* for 10 min at 4 °C), pellets were washed twice with 1 mL of NIB and resuspended in 2 mL of NIB buffer containing 5 μg/mL DAPI. mCherry and DAPI nuclei were sorted on a BD FACSAria III Cell Sorter (70 μm nozzle), and gates were adjusted using Col-0 wild-type samples as a reference. Nuclei were sorted into CTAB buffer (Biochemica #A4150) containing Rnase A (Thermo scientific #R1253). After sorting, the suspension was heated to 60 °C for 10 min, and chloroform/isoamyl alcohol (24:1) was added. Phases were mixed and centrifuged (14000 × *g* for 1 min). The upper aqueous phase was transferred to a new tube with 0.7 volume of cold isopropanol, 150 mM of NaAc, and 1 μL of glycogen (Thermo scientific #R0561) and incubated at −20 °C overnight. The next day, tubes were centrifuged (14000 × *g* for 10 min), and the pellets were washed twice with 70% ethanol. After removing ethanol, pellets were resuspended in 10 μL of nuclease-free TE buffer. DNA concentration was measured using the Picogreen dsDNA Assay Kit (Invitrogen #P11496).

### Sampling and DNA extraction

For Col-0 WT, CS, HS, and mutant *Arabidopsis* plants, harvesting was done from MS agar plates in sterile conditions (3 replicates for each condition). Single plantlets were carefully removed from the agar plates and leaf tissues were harvested in liquid nitrogen. DNA extraction was performed using a CTAB (cetyltrimethylammonium bromide) protocol [41] followed by an ethanol precipitation step. DNA quality was measured on 1% agarose gel and with a NanoDrop spectrophotometer. DNA was quantified using a Quantus Fluorometer (Promega). All the following steps involving handling DNA were performed with wide-bore pipet tips.

### Removing large linear DNA fragments

To remove large genomic linear DNA fragments and small degradation products, 5 μg of genomic DNA was purified using a Geneclean kit (MPBio) according to the manufacturer’s instructions with slight modifications. 5 μg DNA was dissolved in 20 μl water and mixed with 3 volumes (60 μl) NaI 6 M solution. Glassmilk suspension of silica matrix (MPBio), was thoroughly resuspended by vortexing for 1 min and 10 μl glassmilk was added to the DNA-NaI solution. The mixture was gently inverted and incubated at room temperature for 5 min, mixing every 1–2 min. DNA bound to glassmilk was pelleted by centrifugation and the pellet was washed twice with NEW wash solution (MPBio). After washing, the DNA was resuspended in 10 μl water and quantified again on a Quantus Fluorometer (Promega).

### Extrachromosomal circular DNA enrichment

Rolling circle amplification (RCA) was performed to amplify eccDNA as previously described [42] with some modifications. A 20 μl reaction was set up using 20 ng of size-selected template DNA, 1 mM dNTPs, 10 U Phi29 DNA polymerase (Thermo Fisher), 50 μM Exo-resistant random primer (Thermo Fisher), 0.02 U inorganic pyrophosphatase (Thermo Fisher) and 1× Phi29 DNA polymerase buffer (Thermo Fisher). The reaction was run at 30 °C for 18 hr and stopped by heating to 65 °C for 2 min. DNA was precipitated by adding 0.1 volume of 3 M sodium acetate (pH 5.2), 2.5 volumes of ethanol, and 1 μl of glycogen (Thermo Fisher) and incubating overnight at −20 °C.

### Resolving hyperbranched RCA amplicons

A series of DNA debranching, branch-release, and DNA repair reactions were used to resolve the hyperbranched RCA amplicons as described before [43] with some modifications. An amount of 10 μg of RCA amplified DNA was used in a debranching reaction with 5 U of Phi29 DNA polymerase (Thermo Fisher) without primers at 30 °C for 2 h and stopped by heating at 65 °C for 2 min. The product was precipitated with sodium acetate/ethanol as described above. The purified product was treated with 50 U S1 nuclease (Thermo Fisher) in a 20 μl reaction at 37 °C for 30 min and stopped by adding 3.3 μl of 0.5 M EDTA and heating at 70 °C for 10 min. DNA was purified by sodium acetate/ethanol precipitation. Purified DNA was treated with 3 U T4 DNA polymerase (New England Biolabs) and 10 U *Escherichia coli* DNA polymerase I (New England Biolabs) with 1× NEBuffer 2 and 1 mM dNTPs in a 50 μl reaction. The reaction was incubated at 25 °C for 1 h and stopped by heating at 75 °C for 20 min. Dephosphorylation was conducted by adding 5 U of Alkaline Phosphatase (Thermo Fisher) and incubating at 37 °C for 10 min, followed by stopping the reaction by heating at 75 °C for 5 min. The repaired DNA was purified using Agencourt AMPure XP Beads (Beckman Coulter) at a 1.5× volumetric ratio.

### Libraries preparation and PacBio sequencing

A Bioanalyzer 2,100 12K DNA Chip assay (5067-1508, Agilent) was used to assess the fragment size distribution of the enriched DNA samples. The sequencing libraries were produced using the SMRTBell Barcoded Adapter Complete Prep Kit-96, following the manufacturer’s instructions (100-514-900. Pacific Biosciences). Approximately 200 ng of each DNA sample was end-repaired using T4 DNA Polymerase and T4 Polynucleotide Kinase according to the protocol supplied by Pacific Biosciences. A PacBio barcoded adapter was added to each sample via a blunt end ligation reaction. The samples were then pooled together and treated with exonucleases in order to create a SMRT bell template. Library fractions were quality inspected and quantified on the Agilent Bioanalyzer 12 kb DNA Chip and on a Qubit Fluorimeter respectively. A ready-to-sequence SMRTBell-Polymerase Complex was created using the P6 DNA/Polymerase binding kit 2.0 (100-236-500, Pacific Biosciences) according to the manufacturer’s instructions. The Pacific Biosciences RS2 instrument was programmed to load and sequence the samples on SMRT cell v3.0 (100-171-800, Pacific Biosciences), taking one movie of 360 min. A MagBead loading (100-133-600, Pacific Biosciences) method was chosen to improve the enrichment of longer DNA fragments. After the run, a sequencing report was generated for every cell via the SMRT portal to assess the adapter dimer contamination, sample loading efficiency, the obtained average read length, and the number of filtered sub-reads.

### PacBio data analysis

Barcode-separated PacBio subreads were generated as the result of SMRT sequencing. The sequencing data was processed on SMRT Link v8.0 (Pacific Biosciences) for demultiplexing and extraction of circular consensus sequencing (ccs) reads using the following filtering criteria: minimum predicted accuracy = 99.9, and minimum read length of insert (in bases) = 3,000. Processing PacBio data on SMRT Link ccs enables the selection of reads with user-defined accuracy [44]; thus, the same analysis was repeated by changing the minimum predicted accuracy to 99.5 and 99.0 and only the reads with 99.9% accuracy post-ccs were selected for downstream analyses. Post-99.9-ccs PacBio reads (ccs reads) were processed on deconcat (https://github.com/devang-mehta/ciderseq2) as described before [30] and only the reads with more than one round of concatenation were retained, referred in this paper as eccDNA reads. Clustering analysis was performed using cd-hit [45] at default identity and tolerance to generate a non-redundant database.

### EccDNA mapping and annotation

For eccDNA mapping on the *Arabidopsis* genome, Col-0 genome assembly was obtained from TAIR version 10 (https://www.arabidopsis.org/). Mapping was done using BLAST [46] with following parameters; -evalue = 1e-50, -num_threads = 20, -max_hsps = 1, -max_target_seqs = 1,000, -best_hit_overhang = 0.1, -best_hit_score_edge = 0.05 (S10 Table). A BLAST database was generated with all annotated *Arabidopsis* TEs obtained from TAIR version 9 (https://www.arabidopsis.org/) and eccDNAs were mapped using BLAST on this database to obtain percentages of partial and full-length TEs in eccDNAs; where full TEs represent complete TE within eccDNA and partial TE means the eccDNA does not cover the whole TE (eccDNA length < TE length; S9 Table). TE family and superfamily classification was obtained from TAIR (https://www.arabidopsis.org/browse/transposon_families). Mapping on the CDS database was done the same way by utilizing the tair9 cds dataset (S4 Table). Gene ontology analysis was done using ShinyGO 0.77 (http://bioinformatics.sdstate.edu/go/).

### EccDNA quantification and downstream statistical analyses

For eccDNA quantification, chromosomes from *Arabidopsis* genome assembly tair10 were divided into 100 kb windows, and eccDNA reads were mapped to each window using BLAST [46], to obtain eccDNA counts per 100 kb. For downstream comparative analyses, the eccDNA counts were normalized using the formula ‘eccDNA counts per 100 kb window/total eccDNA reads from respective biological replicate × 100’.

Normalization of TE-derived eccDNA was performed using the formula: *Normalized eccDNA abundance = *(*Number of TE-derived eccDNA reads*/*Total number of CCS reads in the sample*) × 100. This normalization approach scales the eccDNA reads to a percentage of the total CCS reads in each sample, which effectively standardizes the data across different sequencing depths and biological replicates. This standardized approach mitigates bias due to the differing total number of reads obtained per sample, and allows for comparisons of eccDNA abundance across samples. Statistical analyses were then performed on these normalized values and this information was used in the downstream comparative analyses such as to calculate standard deviation, indicated by error bars in respective figures. Statistical analyses were conducted using ANOVA or *t*-tests, for the comparison of multiple groups simultaneously or to analyze data involving comparisons between two groups, respectively. Statistical significance was only accepted when the *p*-value < 0.05.

### DNA blot analysis

Southern blots were performed as described previously [47] using an *ONSEN*-specific probe (S2 Table). For eccDNA chr3_14202454 and chr5_3253118, inverse primers were used to amplify the region encompassing the junction of eccDNA, and respective amplicons were used as probes (S2 Table). Ten micrograms of total gDNA and RCA-amplified DNA was digested by restriction with 20 U of PsiI and EcoRI (Thermo Fisher), respectively, in an overnight reaction. DNA was separated on a 1.5% agarose-TAE gel and transferred overnight onto a positively charged nylon membrane (Roche). This was followed by UV-crosslinking and hybridization with the *ONSEN* IR-DIG-labeled probe. DNA bands were visualized using alkaline phosphatase-conjugated anti-DIG (1:10,000) and CPD chemiluminescent substrate.

## Results

### EccDNAs are predominantly derived from centromeric and pericentromeric regions and are enriched in TEs

**Fig 1:**
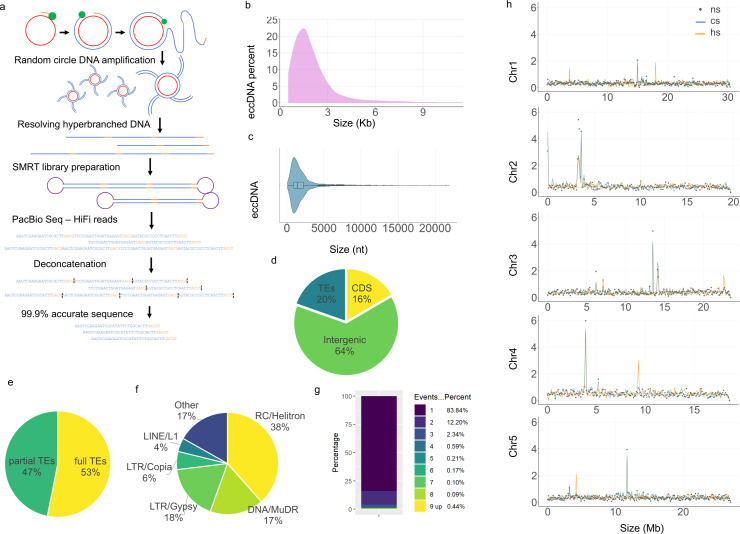
Dynamics and distribution of eccDNA in *Arabidopsis.* **(a)** Schematic representation of eccDNA amplification and sequencing via CIDER-Seq pipeline. **(b)** EccDNA size distribution in *Arabidopsis* Col-0 leaf tissues (*n* = 4,158). The *x*-axis shows eccDNA size in kb and the *y*-axis shows the percentage of eccDNAs belonging to each window. **(c)** Size ranges of eccDNAs with marked median and quantiles (*n* = 4,190). **(d)** Percentages of eccDNA reads mapping to TAIR CDS, TE, or intergenic databases (values represent the average of three replicates). **(e)** Percentages of partial and full-length TEs in eccDNAs (values represent the average of three replicates); where full TEs represent complete TE within eccDNA and partial TE means the eccDNA does not cover the whole TE, i.e., eccDNA length < TE length. **(f)** Percentages of TE-derived eccDNA reads mapping to different *Arabidopsis* TE superfamilies (values represent the average of three replicates). **(g)** Cd-hit clustering analysis indicates that the majority of clusters (83.84%) are single-event clusters (*n* = 9,801). **(h)** EccDNAs mapped to *Arabidopsis* chromosomes. The *x*-axis indicates chromosome size in Mb and the *y*-axis indicates normalized eccDNA reads per 100 kb window. Heat stress (hs), control (cs), and non-stressed (ns) *Arabidopsis* samples (3 replicates for each condition). The raw data supporting all figures can be found in S1 Data.

A comprehensive map of the eccDNA population in *Arabidopsis* was generated using the CIDER-Seq pipeline (Fig 1a) [30] on DNA purified from *Arabidopsis* leaf tissues. In this de-novo assembly of high-accuracy circular DNA molecules, we identified on average 4,675 eccDNAs per sample (S1 Table; S1 Fig) including molecules as large as 22,431 nt (Fig 1b), with an average length of 3,681 nt (median 3,347; Fig 1c). We validated our approach by verifying a previously reported *LTR/Copia* retrotransposon, *ATCOPIA78*/*ONSEN*, that is activated upon heat stress (S2 Fig) [10,48]. Comparative analysis of eccDNA copies in *Arabidopsis* under heat stress (HS; 37 °C for 24 hr), control stress (CS; 4 °C for 24 hr), and no stress (NS; no temperature shift) indicated the accumulation of *ONSEN* eccDNAs upon heat stress (S3a Fig). We further verified a 5-fold increase of *ONSEN* copies in HS compared to CS (S3b Fig), as observed in other studies using independent molecular techniques [10,48] indicating that our eccDNA datasets can be used for quantitative analyses. Comparing the copies of younger and older *ONSEN* eccDNAs indicated that most eccDNAs (73%) are derived from younger *ONSEN* genomic loci (S3c Fig). Thus, the long-read eccDNA datasets allowed us to quantitatively study the population diversity of eccDNA-making TE copies and revealed the association between the evolutionary timescale, mutation accumulation, and TE mobilization. Finally, for additional independent validation of eccDNAs, we selected two previously uncharacterized eccDNA, chr3_14202454; and *VANDAL18NA* (chr5_3253118). By using a probe reverse-complementary to the junction region in chr3_14202454 and chr5_3253118 (S2 Table) in a DNA blot, we detected their presence in *Arabidopsis* leaf tissue (S4 Fig), providing an independent validation of TE mobilization via eccDNA intermediates.

Since there is no formally accepted classification system for plant eccDNAs, we investigated the different types by mapping eccDNA reads on different TAIR datasets, such as coding sequences (CDS), intergenic regions, and TE (Fig 1d). These analyses revealed several interesting insights. Firstly, we identified the enrichment of gene ontologies related to the RNA metabolic processes (S3 and S4 Tables). Secondly, we observed that on average 53% of the TE-mapped eccDNA reads encompassed full-length TEs (Fig 1e, S5 Table). Furthermore, the majority of TE-containing eccDNAs belonged to the *RC/Helitron* (38%), followed by the *DNA/MuDR*, as observed in the TE superfamilies-based eccDNA mapping (Figs 1f; S5; S6 Table). Individual TEs with the most abundant eccDNA reads were also belonging to *RC/Helitron* (S7 Table). Further analysis of *RC/Helitron* indicated that, within the *RC/Helitron* superfamily, the most abundant TE families were *ATREP3, ATREP4, ATREP10*, and *HELITRONY3* (S8 Table). Because helitrons are also the most abundant TEs inserted in the *Arabidopsis* genome [49], we checked if the TE families found in eccDNA are enriched more than expected to their respective composition in the *Arabidopsis* genome. In these analyses, we observed that while helitrons conformed to their expected genomic proportions in the *Arabidopsis* genome, other TE superfamilies*, e.g.*, *LTR/Gypsy* TEs were significantly overrepresented (*p*-value < 0.05). Moreover, the *‘DNA’* TE superfamily, the fourth most abundant TE superfamily in the *Arabidopsis* genome, was not enriched in the eccDNA datasets (Fig 1f; S9 Table). These results, alongside the observations on the varied abundance of TE superfamilies in *Arabidopsis* mutants, suggest that TE distribution in eccDNA datasets does not directly mirror the overall TE composition of the *Arabidopsis* genome.

The clustering analyses of *Arabidopsis* eccDNAs identified 84% of single-event eccDNAs (Fig 1g), where single-event is defined as described by [29]; ‘single continuous genomic loci (continuous eccDNAs, self-circularization of a single genomic fragment)’. Our observation is comparable to the 89% of single-event eccDNAs observed in the mammalian cell lines [29]. Interestingly, the *Arabidopsis* eccDNAs were enriched in the centromeric and pericentromeric regions, as revealed by mapping the eccDNA datasets on *Arabidopsis* genome and evaluating eccDNA abundance per 100 kb bins on *Arabidopsis* chromosomes (Fig 1h, S10 Table). To further investigate the genomic context of eccDNA biogenesis, we specifically analyzed *ATHILA* family elements based on their chromosomal location. Our analysis revealed that a significantly higher number of eccDNAs originated from *ATHILA* elements located within the centromeric regions, compared to those from non-centromeric regions (S6a Fig). This enrichment was particularly striking for *ATHILA6A* and *ATHILA6B* elements, which are highly abundant in the centromeric region of chromosome 5 [50]. Consistent with this, we observed an overrepresentation of these families in eccDNAs derived from chromosome 5 (S6b Fig), indicating that centromeric *ATHILA* elements might be active contributors to eccDNA formation. Together, the generated eccDNA datasets revealed centromeric/pericentromeric regions as hotspots of *Arabidopsis* eccDNAs with several eccDNAs containing full-length TEs.

### DNA methylation selectively regulates the production of EccDNAs from different TE classes

**Fig 2:**
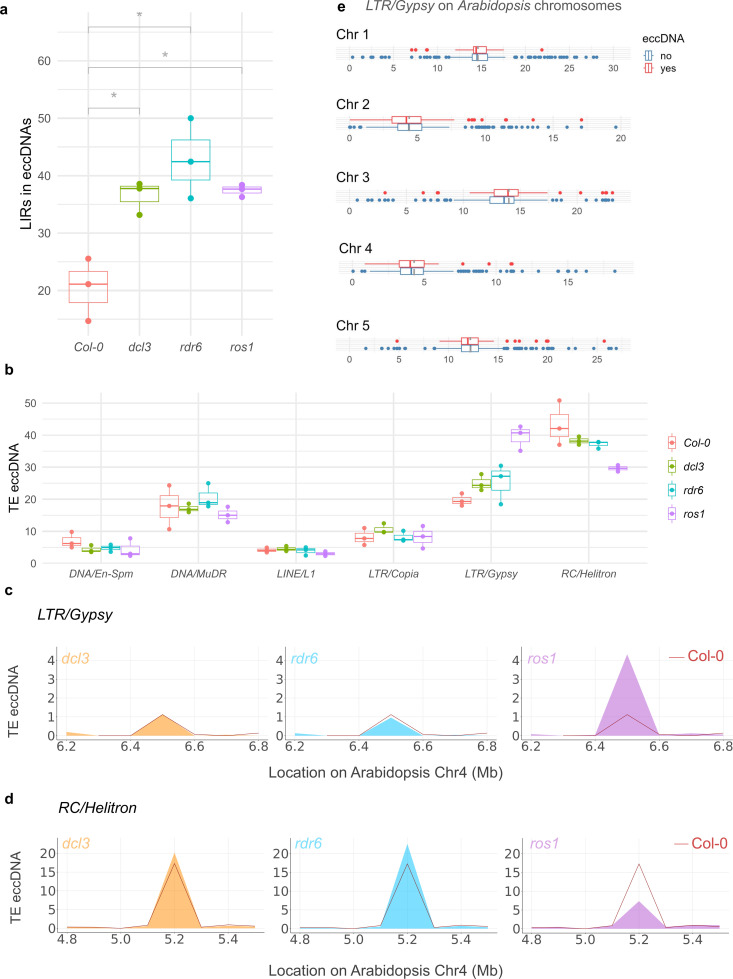
Impaired DNA methylation shifts eccDNA profile. **(a)** Long inverted repeats (LIR) identified in the eccDNAs of Col-0 and mutants *dcl3, ros1,* and *rdr6.* A higher number of LIRs are observed in eccDNAs from *Arabidopsis* methylation mutants, compared to the Col-0. **(b)** EccDNA from TE superfamilies in Col-0 WT and *dcl3*, *rdr6,* and *ros1* mutants. *y*-axis indicates the normalized eccDNAs abundance belonging to each TE superfamily. (c) *LTR/Gypsy*-derived eccDNAs from Col-0 WT and *dcl3*, *rdr6,* and *ros1* mutants mapped on *Arabidopsis* Chr 4 (3 replicates). **(d)** *RC/Helitron* eccDNA from Col-0 WT and *dcl3*, *rdr6,* and *ros1* mutants mapped on *Arabidopsis* Chr 4 (3 replicates). **(e)** Mapping eccDNA-producing and non-eccDNA-producing *LTR/Gypsy* elements identified in *ddm1* and mapped on *Arabidopsis* chromosomes. Gray dashed line indicated the centromere on respective chromosomes and the *x*-axis indicates chromosome size in Mb. No particular enrichment of eccDNA-making *LTR/Gypsy* is observed within or near the protein coding regions. The raw data supporting all figures can be found in S1 Data.

DNA methylation is one of the major mechanisms used by plants to control transposon activity [21]. Since we identified eccDNA-making TEs, we asked if there is a correlation between eccDNA loci identified in this study, DNA methylation (methyl C) [51], and active histone H3 lysine 4 methylation (H3K4me) sites [52]. This analysis indicated no significant correlation between eccDNA activation and methyl C or active H3K4me3 (S7 Fig). Correlation analysis with DNA methylation levels specifically in euchromatin, pericentromeric, and centromeric regions corroborated these results (S8 Fig). Since there can be multiple cellular eccDNA biogenesis mechanisms operating in a methylation-independent manner (e.g., in the case of apoptosis [29]), or methylation-dependent manner (e.g., in the case of TE-derived eccDNAs), we analyzed eccDNAs from independent TE superfamilies/families in representative *Arabidopsis* methylation mutants. Specifically, we performed eccDNA sequencing of *rdr6*, a knock-out of an RNA-dependent RNA polymerase involved in TE silencing [53]; *dcl3*, a mutant lacking 24nt siRNAs-generating dicer involved in RNA-directed DNA methylation (RdDM) [54]; and *ros1*, a knock-out of the *Arabidopsis* demethylase that limits the spread of DNA methylation at TE/repeat boundaries [37]. In these analyses we discovered several eccDNAs affected by RdDM, non-canonical DNA methylation or DNA hypermethylation from *Arabidopsis* TEs belonging to *RC/Helitron* (S9 Fig), *LTR/Copia* (S10 Fig), *LTR/Gypsy* (S11 Fig), *DNA/MuDR* (S12 Fig), *DNA/En-Spm* (S13 Fig) and *LINE/L1* (S14 Fig) superfamilies. Because long inverted repeats (LIR) have been linked to TE activation, we analyzed if LIRs could promote the formation of eccDNAs by examining LIRBase [55]. We observed a significantly high number of LIRs in eccDNAs from methylation mutants compared to the wild-type (Fig 2a). We found that eccDNAs corresponding to LIR structures were more frequently detected in methylation mutants *dcl3, ros1*, and *rdr6*, but not in *ddm1* (S15 Fig). This suggests that eccDNA formation from LIR fragments may rely more on non-CG methylation pathways rather than on the global heterochromatin decompaction observed in *ddm1*.

Previous studies have shown that ROS1 removes methylation from TEs and their neighboring genes, thus, a *ros1* mutant has a hypermethylated genome [56,57]. Our eccDNA profiling of *ros1* indicated an overall increase in *LTR/Gypsy* (Fig 2b and 2c) and a decrease in *RC/Helitron* transposons (Fig 2d), while these TEs remained relatively stable in wt and *dcl3* plants (Fig 2c and 2d). Hyperactivation of *LTR/Gypsy*-eccDNAs in *ros1* mutant was surprising because previous studies have shown that ROS1 preferentially targets TEs closer to protein-coding genes [57] while the *LTR/Gypsy* TEs are enriched in the pericentromeric regions [50]. One explanation could be that the *LTR/Gypsy* TEs identified in *ros1* eccDNA datasets, such as *ATHILA* elements (S6 Fig), belong to a subset of these TEs activated in the absence of ROS1 demethylase and thus could be concentrated in the neighboring regions of protein-coding genes away from the centromeres. However, comparing eccDNA-producing *LTR/Gypsy* elements identified in *ros1* eccDNA datasets with all *LTR/Gypsy* TEs in the *Arabidopsis* genome indicated no particular eccDNA-making *LTR/Gypsy* enrichment in the neighboring regions of protein-coding genes (Fig 2e). These findings suggest a noncanonical TE control by DNA methylation pathways, potentially involving eccDNA biogenesis, warranting further investigation into the specific mechanisms by which ROS1 and other demethylases influence TE eccDNA formation and their potential roles in plant genome regulation.

### Transcriptomic and eccDNA correlations in actively dividing *Arabidopsis* cells

**Fig 3:**
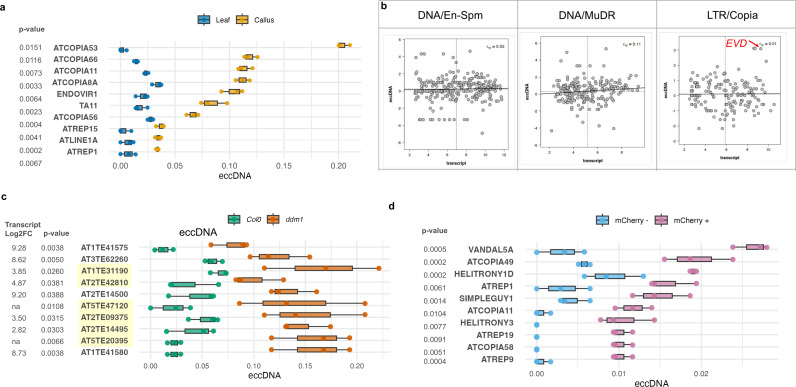
EccDNA in *Arabidopsis* meristem and callus. **(a)** TE-derived eccDNAs differentially regulated in *Arabidopsis* callus compared to leaf tissue with their respective *p*-values. **(b)** TE-derived eccDNAs in *ddm1* mapped to representative TE superfamilies. *EVD LTR/Copia* with high transcript and eccDNA copy number is highlighted in red. **(c)** High copy number TE-derived eccDNAs with their respective *p*-values and transcript log_2_ fold change in *ddm1*. *ATCOPIA93* (*EVD*) and *VANDAL21* have been previously identified in *ddm1* datasets; the newly identified TEs are highlighted. **(d)** TE-derived eccDNAs differentially regulated in shoot apical meristems (mCherry +) compared to surrounding cells (mCherry −) obtained by fluorescence-activated nuclear sorting. The *x*-axis in panels **a, c, and d** indicates normalized eccDNA abundance. The raw data supporting all figures can be found in S1 Data.

To check if differential methylation associated with active cell division could alter eccDNA profiles, we tested the correlation between eccDNA abundance and gene expression in actively replicating pluripotent cells of *Arabidopsis* [58]. First, we generated the eccDNA profile of *Arabidopsis* calli and performed a comparative analysis of the relative eccDNA abundance in calli versus leaf tissues (S16 Fig). We then used a published dataset of *Arabidopsis* transcript fold change in calli versus leaf tissues [59] to investigate a potential correlation with eccDNA induction in calli. Despite a weak correlation between transcript levels and eccDNA in calli (Pearson’s *R* = 0.25; S17 Fig), the analysis revealed calli-enriched eccDNAs, and among them, several eccDNAs originated from *Arabidopsis* TE superfamily *LTR/Copia* (Fig 3a). Of particular interest was an *LTR/Copia* element *ATCOPIA53*, that has been linked previously with the retrotransposon-mediated control of self-incompatibility in polymorphic *A. thaliana* lines [60]. Likewise, *ATCOPIA66’*s noncanonical structure with short internal tandem repeats has been proposed to increase RNAi-mediated chromatin modifications [61]. The eccDNA enrichment and active transcription of those TEs particularly in calli call for further investigation of their potential role in cell differentiation/dedifferentiation.

In the shoot apical meristem (SAM) of *Arabidopsis*, the dynamic transcription and DNA methylation patterns, particularly the transient increase in transposon expression and changes in CHG and CHH methylation, suggest an epigenetic reprogramming that may protect genome integrity in stem cells as they transition into the reproductive lineage, potentially involving eccDNA [40]. We therefore aimed at profiling eccDNAs in *Arabidopsis* SAM-enriched cells obtained using FANS [39,40] from the *Arabidopsis* mutant *ddm1* [62] in which the altered access of DNA methyltransferases to condensed heterochromatin results in TEs activation [63,64]. We generated the eccDNA profiles of SAM and surrounding cells with respective controls (S18 Fig). Next, we utilized the published transcriptomic dataset of *ddm1* [65] and performed correlation analyses between TE eccDNAs and transcripts in TE superfamilies (Fig 3b). As seen in the calli, while no overall correlation was found, we observed several TEs with both high eccDNA copy number and transcript expression in *ddm1* (Figs 3b, 3c, and S19). Among these high copy number eccDNAs was a *Ty1/Copia* element *ATCOPIA93 Évadé* (*EVD*) (Fig 3b) that has been reported to be reactivated and mobilized upon release of epigenetic silencing in *ddm1* mutant background [63,64]. Of note, these analyses also led us to identify new TEs that have not been characterized in previous *ddm1* studies [19], such as *VANDAL2* that belong to a subset of TEs defined as variable TEs (Fig 3c). ‘Variable TEs’, as defined by Dubin and colleagues, refer to a specific subset of TEs identified by their dynamic methylation patterns in response to environmental temperature fluctuations. These elements exhibit elevated CHH methylation at higher temperatures and are characterized by their euchromatic localization, high expression levels, and recent insertion into the genome, suggesting a role in rapid genomic adaptation to environmental changes [66]. Identification of variable TEs, therefore, provides a unique insight into the interaction between eccDNA, epigenetic modifications and environmental response in plants.

Finally, by focusing the analysis on TEs displaying significant eccDNA activation and transcriptional up-regulation, we could identify SAM-enriched TE eccDNAs (Figs 3d and S19). In these analyses, we identified several TEs detected as eccDNAs specifically in SAM such as *ATREP19* and *ATCOPIA58*. Interestingly, like *VANDAL2, ATREP19* has also been characterized as variable TE, indicating that the several properties associated with the variable TEs, particularly differential CHH methylation, may be linked to the eccDNA biogenesis. Likewise, *ATCOPIA58* insertion and methylation state was detected in MethylC-seq data by the epiTEome program previously designed to analyze DNA methylation specifically in the context of TEs [67]. Because the *Arabidopsis* genome contains a small population of *ATCOPIA58* elements, with only one putatively autonomous copy and four deletion derivatives [68], its specific detection in SAM might reflect altered epigenetic states or chromatin accessibility associated with the unique epigenetic landscape of stem cell-enriched tissues. Together, these findings suggest that eccDNA biogenesis in actively dividing and stem cell-enriched tissues reflects an interplay between TE structure, transcriptional activity, and dynamic epigenetic regulation.

## Discussion

EccDNAs have been identified as ubiquitous elements in several eukaryotes such as yeast, ciliates, amphibians, plants, birds, and mammals including human cells [69,70]. We took advantage of the long-read circular DNA sequencing technology, CIDER-Seq, to explore the eccDNA landscape in various tissues and conditions in *Arabidopsis*. Identification of well-characterized active *ONSEN* TE in heat-stressed *Arabidopsis* indicated that our method is efficient in capturing and quantifying actively proliferating eccDNAs. Chromosome mapping and database clustering analyses of the eccDNAs indicated centromeric/pericentromeric regions as hotspots for *Arabidopsis* eccDNAs.

Analysis of eccDNA datasets revealed the enrichment of coding genes among the eccDNAs datasets. A probable explanation is the near-random origination of eccDNAs from the entire genome, as observed in human eccDNA studies [29] and eccDNA studies on the less repeat-rich genome of model *C. livia domestica* [25]. EccDNAs can arise from diverse sources, including organellar DNA (chloroplast/mitochondrial), TEs, and tRNAs, and recently identified circularized apoptotic DNA fragments with potential immune signaling roles were also recently identified [29]. Previous studies in animals have already shown that a fraction of eccDNAs contain sequences from coding genes [25,29]. Although the possibility of these eccDNAs being an RCA artifact cannot be entirely excluded, it should be noted that RCA-independent sequencing of eccDNA using direct tagging of purified eccDNAs with Tn5 transposase has confirmed the genomic origin of these eccDNAs [29].

The classification of the eccDNAs based on their respective TE superfamilies indicated that a majority of detected eccDNAs belonged to *RC/Helitron* (Fig 1f). We also identified a significantly high LIR abundance in *Arabidopsis* methylation mutants indicating that abolishing LIR silencing could lead to TE activation and eccDNA biogenesis (Fig 2a) [71,72]. Moreover, eccDNA profiling in calli and meristem identified several calli and meristem-enriched eccDNA such as the ones originating from TE superfamily *LTR/Copia* (Fig 3) suggesting that rapid cell multiplication might release epigenetic suppression of some TEs, whose active replication could be detected as eccDNA intermediates. This supports the observations made in previous studies that indicated meristem-specific expression of certain TEs [39,40], and indicates that several eccDNAs identified in *Arabidopsis* tissues (e.g., callus, meristem; Fig 3) are capable of mobilization under varying conditions.

DNA and histone methylation regulates the transcription and transposition of TEs as well as triggers transcriptional silencing of certain genes carrying TEs/repeats in their vicinity [73,74]. In *Arabidopsis*, ROS1 is the active demethylase that prunes siRNA-dependent or -independent DNA methylation at thousands of loci thereby limiting the spread of DNA methylation at TE/repeat boundaries [57]. Our analysis revealed a significant hyperactivation of *LTR/Gypsy*-derived eccDNAs in *ros1* mutant datasets compared to both Col0 controls and other mutants examined. A prior studies demonstrated ROS1 preferentially targets TEs positioned near protein-coding genes [57], while *LTR/Gypsy* elements are typically enriched in pericentromeric regions. One of the possible explanations was that the *LTR/Gypsy* TEs identified within the ros1 eccDNA datasets might represent a subpopulation specifically activated in the absence of ROS1 demethylase activity. This activation could preferentially occur for the *LTR/Gypsy* TEs located nearby protein-coding genes. However, a comparison of eccDNA-producing *LTR/Gypsy* elements within *ros1* eccDNA datasets with the entire *LTR/Gypsy* complement in the *Arabidopsis* genome revealed no specific enrichment of eccDNA-forming *LTR/Gypsy* elements near protein-coding genes. These findings, coupled with previous reports highlighting the critical role of ROS1 in regulating methylation of neighboring genes, prompt further investigation into the complexities of TE/gene regulation by DNA methylation, particularly regarding the specific functions of demethylases like ROS1. Within *ros1*, we also identified several *LTR/Gypsy* loci with significantly increased levels of TE-derived eccDNAs, such as from *LTR/Gypsy* family *ATGP2N*. Recent studies have shown that chromomethylase mutant *cmt3* can reduce TE-derived eccDNAs and transcripts in an antagonistic mechanism with CMT2, i.e., CMT3 can down-regulate TE expression and eccDNA copy number by competing with CMT2 for histone occupancy to perform H3K9me2 methylation [75]. Further profiling studies of *ros1* will be necessary to elucidate whether the control of TE-derived eccDNAs results from a difference in DNA/histone methylation or from other ROS1-operated functions.

EccDNA diversity in the methylation mutant *ddm1* was further explored by comparing our datasets with the latest published dataset [19]. While both datasets revealed the presence of *EVADE* and *VANDAL21* elements in *ddm1* mutants (Fig 3c), our profiling of the *ddm1* eccDNA population exhibited a broader spectrum compared to Zhang and colleagues [19]. This can be explained the methodological differences between the two studies. While our study employed CIDER-Seq, which used high-fidelity PacBio long-read sequencing coupled with the Deconcat algorithm, Zhang and colleagues, employed Illumina and Oxford Nanopore Technologies (ONT)-based mobilome-seq. Among the newly identified eccDNAs were the TEs recognized in a previous study as variable TEs, such as *VANDAL2*. Furthermore, we identified uncharacterized TEs like *ATREP19* (also a variable TE) with high eccDNA in the SAM. This suggests a potential link between eccDNA biogenesis and the unique properties of variable TEs, particularly their temperature-dependent methylation [67]. Our analysis of *ATHILA*-derived eccDNAs revealed that centromeric insertions of *ATHILA* elements disproportionately contribute to eccDNA formation. This observation is in line with previous findings that *ATHILA* elements located within the centromeric regions of chromosome 5 are structurally intact and evolutionarily younger compared to those located outside the centromeres [50]. These younger insertions likely retain intact *cis-*acting features necessary for reverse transcription or circular DNA formation, reminiscent of our observations with the *ONSEN* family, where eccDNAs predominantly originated from younger, transcriptionally active copies. While *A. thaliana* (Col-0) harbors relatively few centrophilic TEs, recent studies in the outcrossing species *Arabidopsis lyrata* have shown a higher abundance of centromeric TEs such as *ALE* [76]. Exploring eccDNA landscapes in *A. lyrata* could therefore provide further insights into the role of centrophilic TEs in shaping eccDNA biogenesis and genome evolution.

In conclusion, we provide a framework for reference-free eccDNA sequencing. This can be particularly useful for species whose genomes have not yet been fully sequenced and accurately annotated. Using various eccDNA datasets, our study revealed differential TE mobilization under varying conditions and genetic backgrounds. It is reasonable to speculate that eccDNA origination could be controlled by different mechanisms, differs between cellular conditions and even varies from cell to cell. Therefore, single-cell profiling of eccDNAs, enabled by selective phi29-based amplification of circular DNA molecules, will also likely help to further characterize and understand the roles of eccDNAs in plants. Finally, comparing TE-specific eccDNAs, we identified several eccDNA-making TEs and demonstrated that the generated eccDNA datasets are valuable resources to study potentially intact and transcriptionally active transposons in *Arabidopsis*. Given the high redundancy of TEs in the genome, there is also a need to develop an artificial TE system to better study the activation and mobilization of eccDNAs in plants, as recently implemented in mammals [77,78]. This information could be key for biotechnology and plant breed applications as TEs can be selectively activated to increase genomic diversity and select novel agronomic traits [79,80].

## Supporting information

S1 Fig Distribution and size range of eccDNAs in heat and cold stress conditions. **(a)** Size range-based distribution of eccDNAs from *Arabidopsis*; where the *y*-axis indicates the normalized eccDNA counts and the *x*-axis indicates the eccDNA size in nucleotides (nt). **(b)** Size distribution in different conditions where boxplots indicate quantiles and medians. NS = Col-0 non-stressed, CS = control stress, HS = heat stress (3 replicates for each condition). The raw data supporting all figures can be found in S1 Data.
(TIFF)

S2 Fig Chromosomal locations and structure of *ONSEN.* **(a)** Chromosomal location of *ONSEN* copies in *Arabidopsis* genome (red triangles); centromeres are marked with black bars. **(b)** Structure of *ONSEN* with LTRs (blue) and location of primers used for amplification (black arrows). **(c)** Circular PCR amplification of ONSEN eccDNA with primers ONSEN_F2 and ONSEN_R1 on genomic DNA of *Arabidopsis* Col-0 wt and mutants *dcl3*, *rdr6*, and *ros1*.
(TIFF)

S3 Fig Heat-stress-induced eccDNA copies of the LTR retrotransposon *ONSEN.* **(a)** Chromosome mapping of eccDNAs originating from heat stress (hs), control (cs), and non-stressed (ns) *Arabidopsis* samples (3 replicates for each condition). Red triangles indicate *ONSEN* eccDNA peaks. **(b)** Relative abundance of eccDNAs containing heat-stress induced *ONSEN* retrotransposon in heat-stress (hs), control (cs), and non-stressed (ns) *Arabidopsis* samples (3 replicates for each condition). **(c)** Relative abundance of younger and older *ONSEN* eccDNA copies with younger *ONSEN* are highlighted in red. The raw data supporting all figures can be found in S1 Data.
(TIFF)

S4 Fig Southern blot and inverse PCR detection of eccDNAs. Southern blot of gDNA (1) and EcoRI-digested Phi29-amplified DNA (2) of Col-0 plants, hybridized with eccDNAs chr3_14202454 and chr5_3253118 probes.
(TIFF)

S5 Fig eccDNAs abundance in *Arabidopsis* TE superfamilies. *Arabidopsis* TE superfamilies are obtained from TAIR. The *y*-axis represents an averaged number of TEs/ total eccDNAs in the sample * 100. NS = Col-0 non-stressed, CS = control stress, HS = heat stress (3 replicates for each condition). The raw data supporting all figures can be found in S1 Data.
(TIFF)

S6 Fig Centromeric localization and chromosome-specific contribution of ATHILA-derived eccDNAs in *Arabidopsis.* **(a)** Distribution of ATHILA transposable elements (TEs) across different families based on their genomic location in centromeric versus non-centromeric regions. Centromeric ATHILA copies were significantly more abundant compared to non-centromeric copies, particularly for ATHILA6A and ATHILA6B families. **(b)** Chromosomal contribution of ATHILA-derived eccDNAs across the five *Arabidopsis* chromosomes. Stacked bar chart shows the number of eccDNA reads mapped to individual ATHILA families per chromosome. Notably, Chromosome 5 contributed the highest number of ATHILA-derived eccDNAs, predominantly driven by ATHILA6A and ATHILA6B families. The raw data supporting all figures can be found in S1 Data.
(TIFF)

S7 Fig Correlation between eccDNAs and DNA methylation. **(a)** Correlation analysis between MethylC and H3K4me3. **(b)** Correlation between eccDNAs and MethylC. **(c)** Correlation between eccDNAs and H3K4me3. R2 indicates the squared value of the correlation coefficient. The units on the *x* and *y* axes are percentage H3K4me3, MethylC or eccDNA where each dot represents the respective percentages of the two parameters on each axis. eccDNA refers to the percentage of eccDNAs from the specific genomic locus represented by the dot, where the corresponding value of the *y*-axis represents the percentage of MethylC (panel b) or H3K4me3 (panel c). The raw data supporting all figures can be found in S1 Data.
(TIFF)

S8 Fig eccDNA distribution compared to the cytosine methylation in Chr1 of *Arabidopsis.* eccDNA distribution and C methylation across chromosome 1; the *x*-axis indicates the location on chromosome (Mb), and the *y*-axis indicates the percentage of eccDNAs and methylated Cs in each window (3 replicates).
(TIFF)

S9 Fig eccDNA profile of *Arabidopsis* TE superfamily *RC/Helitron* in Col-0 wt, and mutants *dcl3*, *rdr6,* and *ros1.* Col-0, *dcl3*, *rdr6*, and *ros1* leaf tissues were processed on the CIDER-Seq pipeline (three replicates for each plant type). *RC/Helitron*-derived eccDNA reads are depicted on the *Arabidopsis* genome; the *y*-axis in each panel indicates the normalized eccDNA reads mapped per 100 kb bins on *Arabidopsis* chromosomes. The genomic region on Chr4, represented in Fig 3c, has been highlighted with a red asterisk.
(TIFF)

S10 Fig eccDNA profile of *Arabidopsis* TE superfamily *LTR/Copia* in Col-0 wt, and mutants *dcl3, rdr6,* and *ros1.* Col-0, *dcl3*, *rdr6*, and *ros1* leaf tissues were processed on the CIDER-Seq pipeline (three replicates for each plant type). *LTR/Copia*-derived eccDNA reads are depicted on the *Arabidopsis* genome; the *y*-axis in each panel indicates the normalized eccDNA reads mapped per 100 kb bins on *Arabidopsis* chromosomes.
(TIFF)

S11 Fig eccDNA profiles of *Arabidopsis* TE superfamily *LTR/Gypsy* in Col-0 wt, and mutants *dcl3, rdr6,* and *ros1.* Col-0, *dcl3*, *rdr6*, and *ros1* leaf tissues were processed on the CIDER-Seq pipeline (three replicates for each plant type). *LTR/Gypsy*-derived eccDNA reads are depicted on the *Arabidopsis* genome; the *y*-axis in each panel indicates the normalized eccDNA reads mapped per 100 kb bins on *Arabidopsis* chromosomes. The genomic region on Chr4, represented in Fig 3b, has been highlighted with a red asterisk.
(TIFF)

S12 Fig eccDNA profiles of *Arabidopsis* TE superfamily *DNA/MuDR* in Col-0 wt, and mutants *dcl3, rdr6,* and *ros1.* Col-0, *dcl3*, *rdr6*, and *ros1* leaf tissues were processed on the CIDER-Seq pipeline (three replicates for each plant type). *DNA/MuDR*-derived eccDNA reads are depicted on the *Arabidopsis* genome; the *y*-axis in each panel indicates the normalized eccDNA reads mapped per 100 kb bins on *Arabidopsis* chromosomes.
(TIFF)

S13 Fig eccDNA profiles of *Arabidopsis* TE superfamily *DNA/En-Spm* in Col-0 wt, and mutants *dcl3, rdr6,* and *ros1.* Col-0, *dcl3*, *rdr6*, and *ros1* leaf tissues were processed on the CIDER-Seq pipeline (three replicates for each plant type). *DNA/En-Spm*-derived eccDNA reads are depicted on the *Arabidopsis* genome; the *y*-axis in each panel indicates the normalized eccDNA reads mapped per 100 kb bins on *Arabidopsis* chromosomes.
(TIFF)

S14 Fig eccDNA profiles of *Arabidopsis* TE superfamily *LINE/L1* in Col-0 wt, and mutants *dcl3, rdr6,* and *ros1.* Col-0, *dcl3*, *rdr6*, and *ros1* leaf tissues were processed on the CIDER-Seq pipeline (three replicates for each plant type). *LINE/L1-*derived eccDNA reads are depicted on the *Arabidopsis* genome; the *y*-axis in each panel indicates the normalized eccDNA reads mapped per 100 kb bins on *Arabidopsis* chromosomes.
(TIFF)

S15 Fig Long inverted repeats (LIR) identified in the eccDNAs across different genetic backgrounds and conditions. The eccDNAs are expressed as % of total LIR-derived eccDNAs along *y*-axis, across different genetic backgrounds and experimental conditions (*x*-axis). Each dot represents an individual biological replicate (*n* = 3 per condition). EccDNAs corresponding to LIRs were enriched in methylation mutants (*dcl3, rdr6, ros1*) but not in *ddm1*. The raw data supporting all figures can be found in S1 Data.
(TIFF)

S16 Fig eccDNA profiles of *Arabidopsis* calli and leaf tissues. DNA extracted from calli induced from the Col-0 leaves was processed on the CIDER-Seq pipeline (three replicates for each condition). eccDNA reads from callus and leaf were mapped on the *Arabidopsis* genome; the *y*-axis in each panel indicates the normalized eccDNA reads mapped per 100 kb bins on *Arabidopsis* chromosomes. The raw data supporting all figures can be found in S1 Data.
(TIFF)

S17 Fig Correlation between eccDNAs and transcript fold change in calli and meristem from *ddm1* mutant. **(a)** Correlation analysis between transcript fold change calli/leaf and eccDNA fold change calli/leaf. The right panel indicates the same analysis on the clustered area. **(b)** Correlation analysis between transcript fold change meristem/non-meristem cells and eccDNA fold change meristem/non-meristem cells in *Arabidopsis ddm1*. The raw data supporting all figures can be found in S1 Data.
(TIFF)

S18 Fig eccDNA profiles of *ddm1* shoot apical meristem-enriched and non-enriched samples. Shoot apical meristem (SAM) cells were sorted using fluorescence-activated nuclear sorting (FANS) and processed, with respective controls from *ddm1* seedlings, on the CIDER-Seq pipeline for eccDNA amplification (three replicates for each cell type). EccDNA reads from SAM (mCherry +), surrounding cells (mCherry −), and *ddm1* seedlings were mapped on the *Arabidopsis* genome; the *y*-axis in each panel indicates the normalized eccDNA reads mapped per 100 kb bins on *Arabidopsis* chromosomes. The raw data supporting all figures can be found in S1 Data.
(TIFF)

S19 Fig Genomic locations of eccDNA-producing TEs highlighted in **Fig 3**. Schematic representation of *Arabidopsis* chromosomes 1–5. Grey bars represent chromosomes, with black segments indicating centromeres. Arrowheads indicate the genomic positions of TEs shown in Fig 3, with colors corresponding to the sample in which their eccDNAs were detected: callus (orange), meristem (blue), and *ddm1* (green). Chromosome coordinates are shown in megabases (Mb).
(TIFF)

S1 Table PacBio sequencing of eccDNA; data, read statistics, and clustering. Demultiplexed data from Sequel II is presented in Mb with the number of reads in corresponding samples; demultiplexing is performed with LIMA. Clustering is performed with cd-hit where unique clusters represent the clusters with single reads. CCS = circular consensus sequencing, NS = Col-0 non-stressed, CS = control stress, HS = heat stress.
(DOCX)

S2 Table Primers used in this study. (DOCX)

S3 Table Gene ontology of eccDNA. (DOCX)

S4 Table EccDNA mapped on *Arabidopsis* CDS. EccDNAs from Col-0, heat-stress, control-stress, meristem-enriched, meristem non-enriched, callus tissues and mutants dcl3, rdr6, ros1, and ddm1 have been mapped on *Arabidopsis* CDS. A BLAST database was generated using the tair9 CDS dataset and mapping was done using BLAST. Various summary statistics are provided such as alignment length, start and end sites, percentage identities, etc.
(XLSX)

S5 Table eccDNA characterization and the number of full-length TEs in eccDNAs. (DOCX)

S6 Table TE-derived eccDNAs from respective TE superfamilies in *Arabidopsis* col-0 and mutants. (DOCX)

S7 Table TEs with most abundant eccDNA reads. (DOCX)

S8 Table TE-derived eccDNAs from respective TE families in *Arabidopsis* col-0 and mutants. (DOCX)

S9 Table EccDNA mapped on *Arabidopsis* transposable elements. EccDNAs from Col-0, heat-stress, control-stress, meristem-enriched, meristem non-enriched, callus tissues, and mutants dcl3, rdr6, ros1, and ddm1 have been mapped on *Arabidopsis* transposable elements (TEs). A BLAST database was generated using the tair9 TE dataset and mapping was done using BLAST. Various summary statistics are provided such as alignment length, start and end sites, percentage identities, etc. TEs selected for transcript abundance analyses in (Fig 3) are highlighted.
(XLSX)

S10 Table EccDNA mapped on *Arabidopsis* genome. EccDNAs from Col-0, heat-stress, control-stress, meristem-enriched, meristem non-enriched, callus tissues, and mutants dcl3, rdr6, ros1, and ddm1 have been mapped on *Arabidopsis* genome (tair10). Mapping was done using BLAST and various summary statistics are provided such as alignment length, start, and end sites, percentage identities, etc.
(XLSX)

S1 Data The raw data supporting all figures. (XLSX)

S1 Raw Images The original, uncropped and minimally adjusted images supporting all blot and gel results. (PDF)

## Abbreviations

ccs: circular consensus sequencing
eccDNA: extrachromosomal circular DNA
FANS: fluorescence-activated nuclear sorting
RCA: rolling circle amplification
SAM: shoot apical meristem
TE: transposable element
