# Supplementary figures and images for: A comprehensive atlas of full-length Arabidopsis eccDNA populations identifies their genomic origins and epigenetic regulation
Source: PLoS Biol. 2025 Jul 15;23(7):e3003275. doi: 10.1371/journal.pbio.3003275 (PMC12273906; doi:10.1371/journal.pbio.3003275)

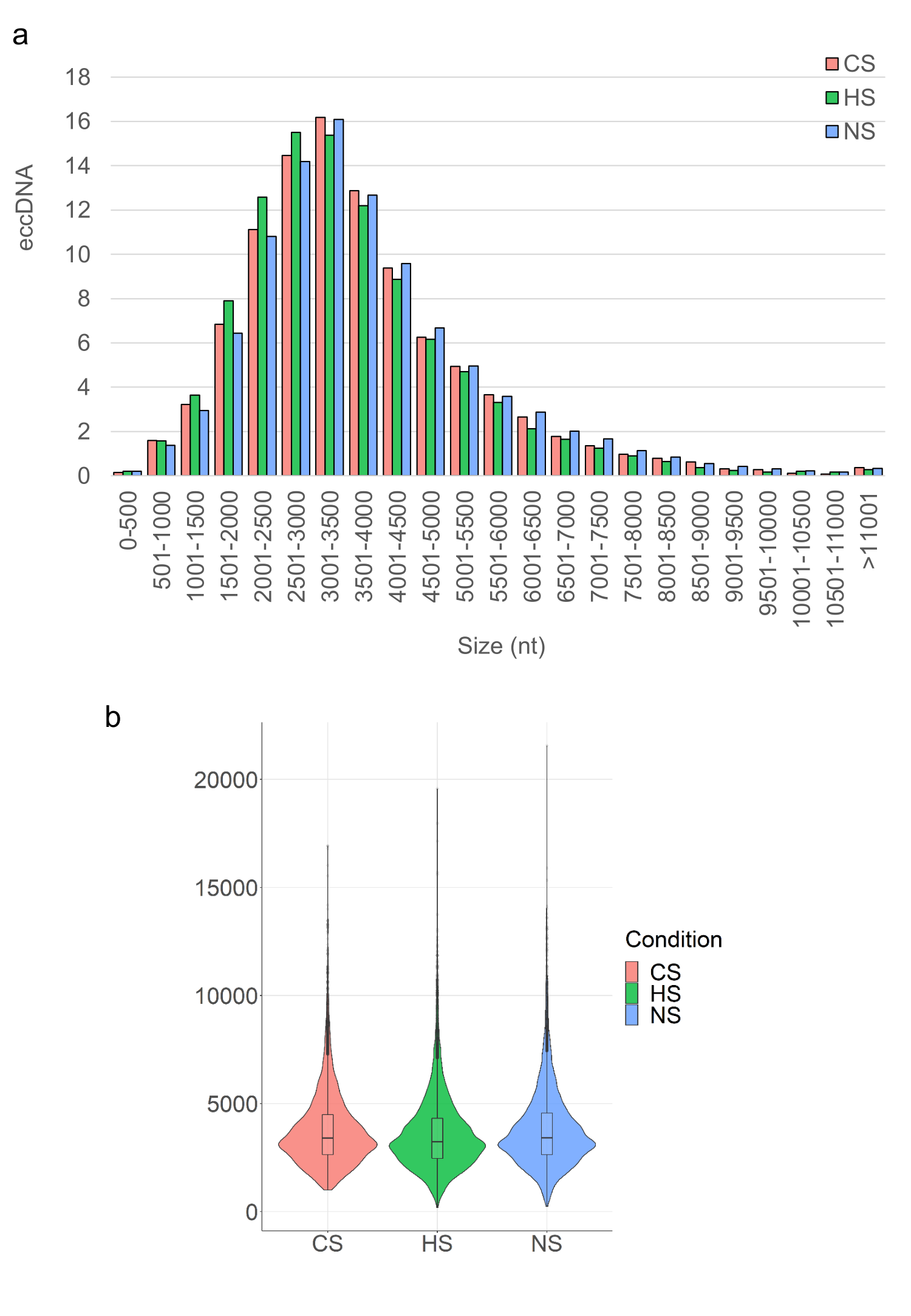

Supplement: S1 Fig — (a) Size range-based distribution of eccDNAs from Arabidopsis; where the y-axis indicates the normalized eccDNA counts and the x-axis indicates the eccDNA size in nucleotides (nt). (b) Size distribution in different conditions where boxplots indicate quantiles and medians. NS = Col-0 non-stressed, CS = control stress, HS = heat stress (3 replicates for each condition). The raw data supporting all figures can be found in S1 Data. (TIFF) [file pbio.3003275.s001.tiff]

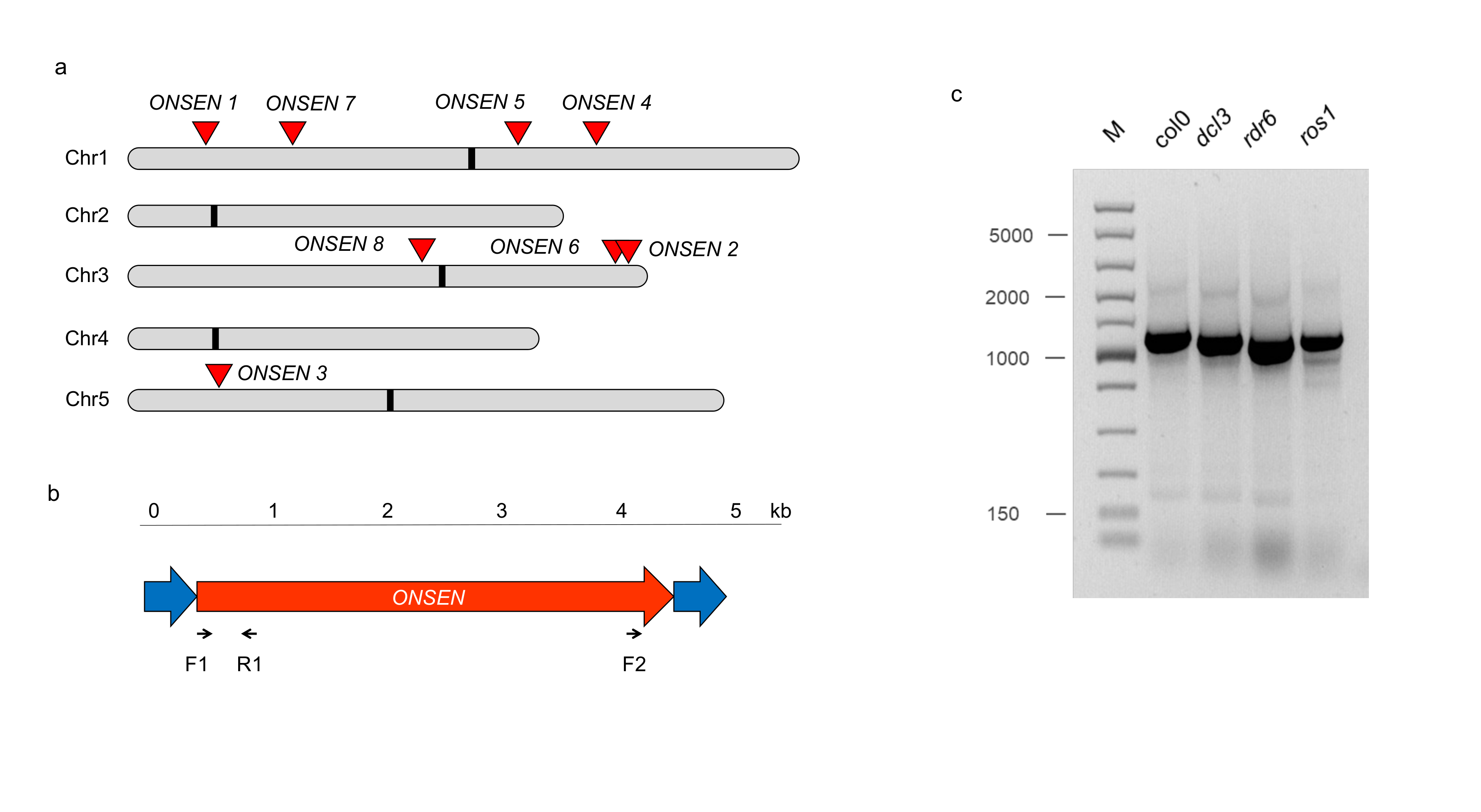

Supplement: S2 Fig — (a) Chromosomal location of ONSEN copies in Arabidopsis genome (red triangles); centromeres are marked with black bars. (b) Structure of ONSEN with LTRs (blue) and location of primers used for amplification (black arrows). (c) Circular PCR amplification of ONSEN eccDNA with primers ONSEN_F2 and ONSEN_R1 on genomic DNA of Arabidopsis Col-0 wt and mutants dcl3, rdr6, and ros1. (TIFF) [file pbio.3003275.s002.tiff]

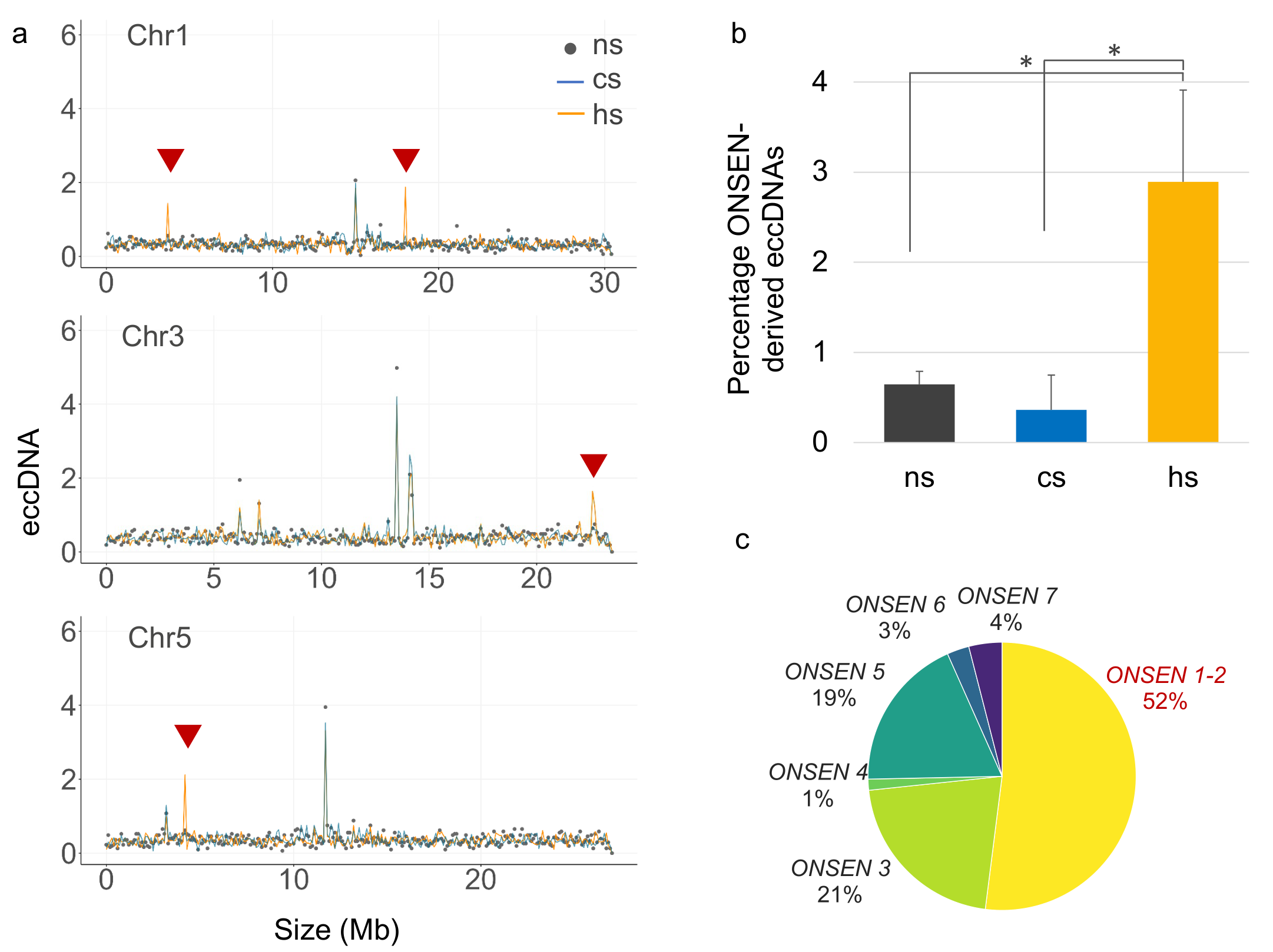

Supplement: S3 Fig — (a) Chromosome mapping of eccDNAs originating from heat stress (hs), control (cs), and non-stressed (ns) Arabidopsis samples (3 replicates for each condition). Red triangles indicate ONSEN eccDNA peaks. (b) Relative abundance of eccDNAs containing heat-stress induced ONSEN retrotransposon in heat-stress (hs), control (cs), and non-stressed (ns) Arabidopsis samples (3 replicates for each condition). (c) Relative abundance of younger and older ONSEN eccDNA copies with younger ONSEN are highlighted in red. The raw data supporting all figures can be found in S1 Data. (TIFF) [file pbio.3003275.s003.tiff]

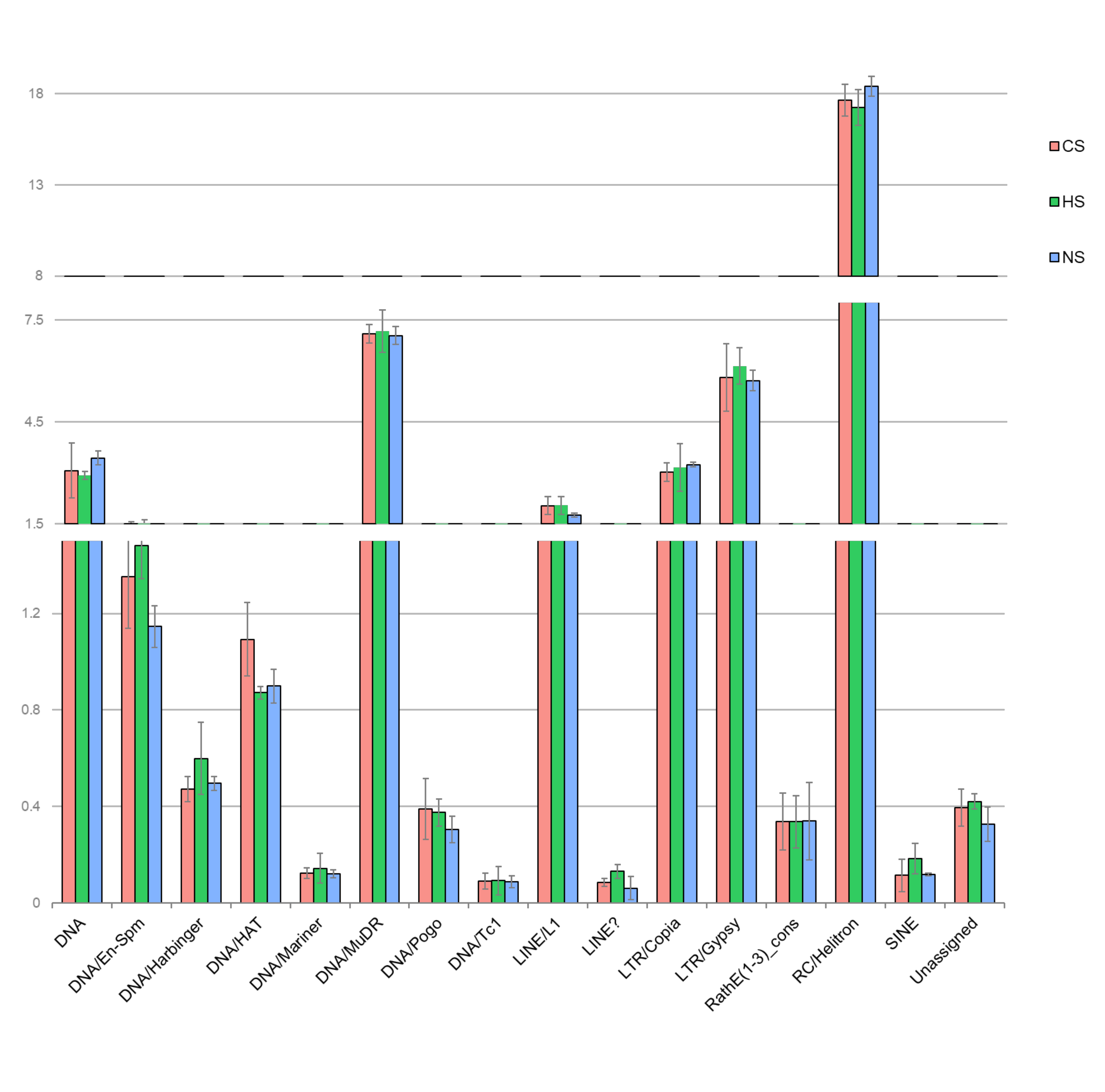

Supplement: S5 Fig — Arabidopsis TE superfamilies are obtained from TAIR. The y-axis represents an averaged number of TEs/ total eccDNAs in the sample * 100. NS = Col-0 non-stressed, CS = control stress, HS = heat stress (3 replicates for each condition). The raw data supporting all figures can be found in S1 Data. (TIFF) [file pbio.3003275.s005.tiff]

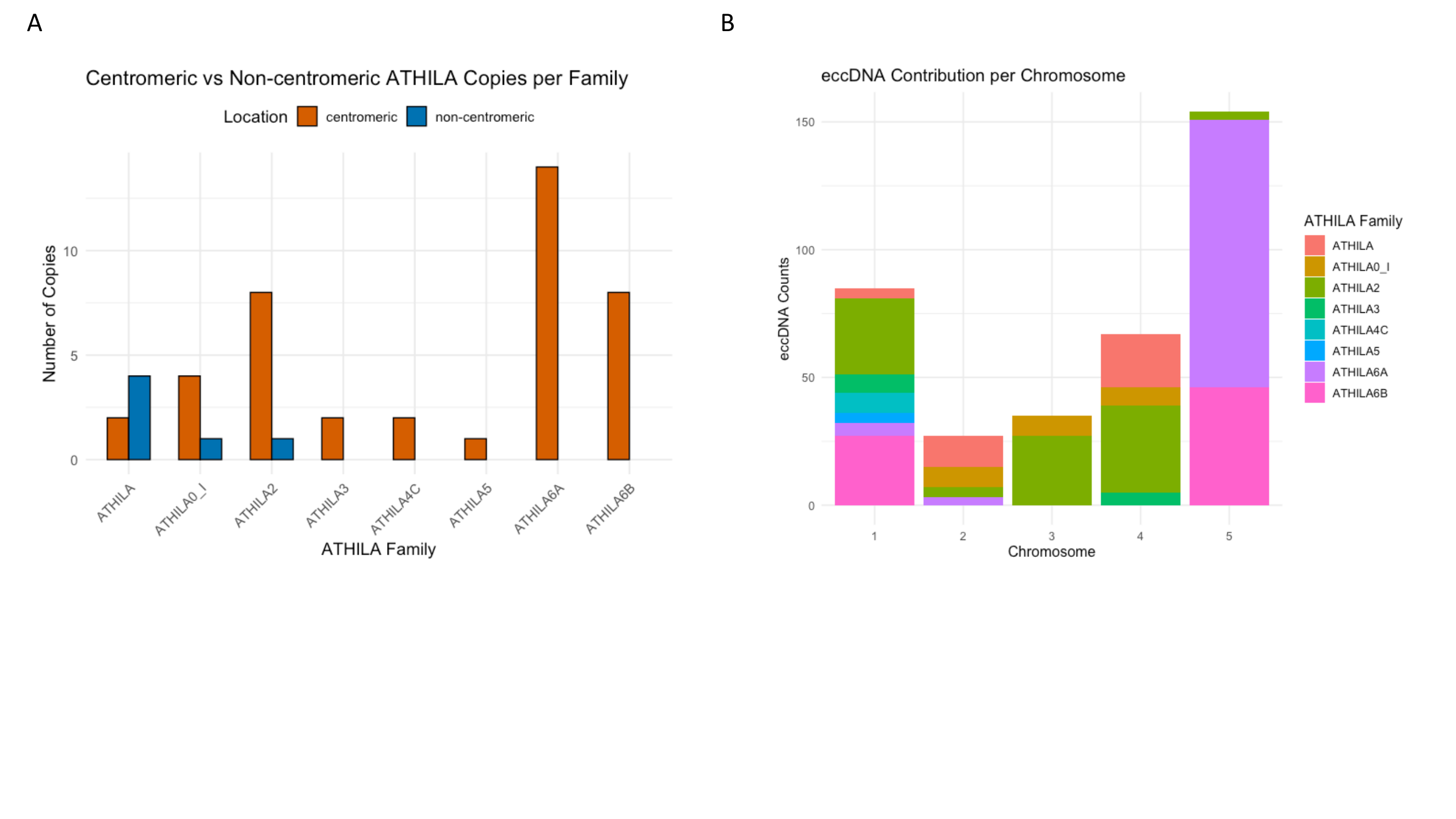

Supplement: S6 Fig — (a) Distribution of ATHILA transposable elements (TEs) across different families based on their genomic location in centromeric versus non-centromeric regions. Centromeric ATHILA copies were significantly more abundant compared to non-centromeric copies, particularly for ATHILA6A and ATHILA6B families. (b) Chromosomal contribution of ATHILA-derived eccDNAs across the five Arabidopsis chromosomes. Stacked bar chart shows the number of eccDNA reads mapped to individual ATHILA families per chromosome. Notably, Chromosome 5 contributed the highest number of ATHILA-derived eccDNAs, predominantly driven by ATHILA6A and ATHILA6B families. The raw data supporting all figures can be found in S1 Data. (TIFF) [file pbio.3003275.s006.tiff]

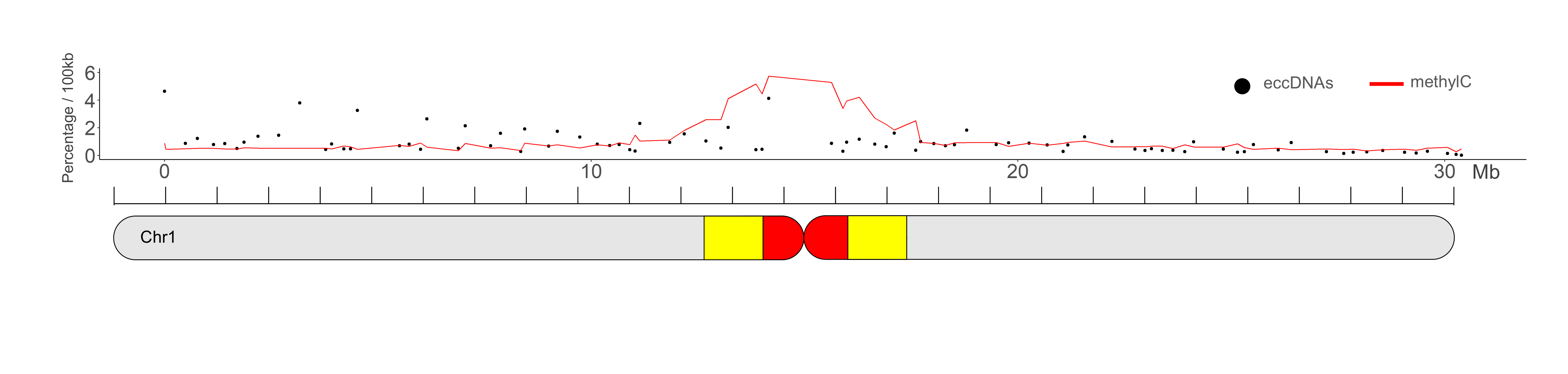

Supplement: S8 Fig — eccDNA distribution and C methylation across chromosome 1; the x-axis indicates the location on chromosome (Mb), and the y-axis indicates the percentage of eccDNAs and methylated Cs in each window (3 replicates). (TIFF) [file pbio.3003275.s008.tiff]

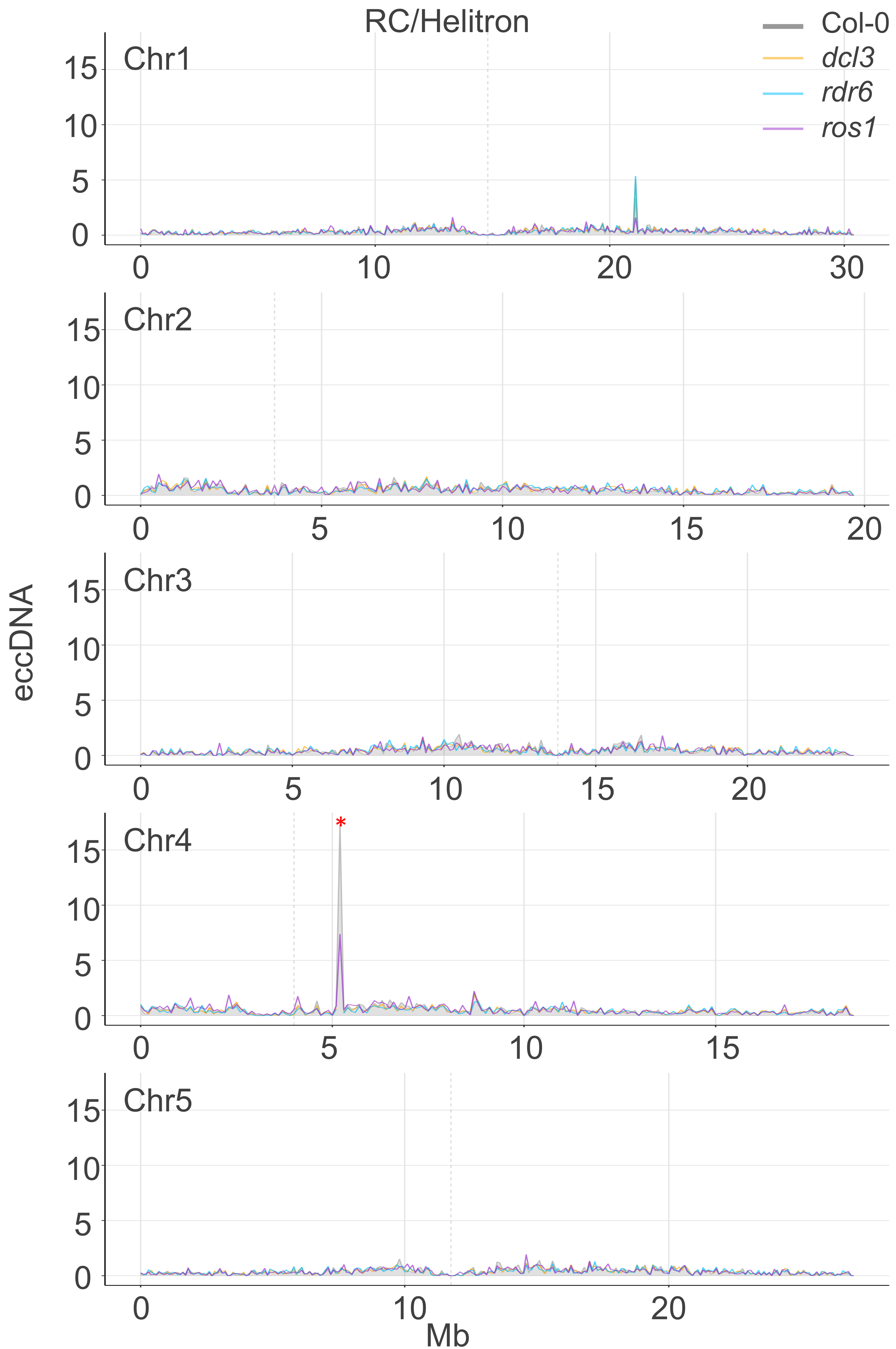

Supplement: S9 Fig — Col-0, dcl3, rdr6, and ros1 leaf tissues were processed on the CIDER-Seq pipeline (three replicates for each plant type). RC/Helitron-derived eccDNA reads are depicted on the Arabidopsis genome; the y-axis in each panel indicates the normalized eccDNA reads mapped per 100 kb bins on Arabidopsis chromosomes. The genomic region on Chr4, represented in Fig 3c, has been highlighted with a red asterisk. (TIFF) [file pbio.3003275.s009.tiff]

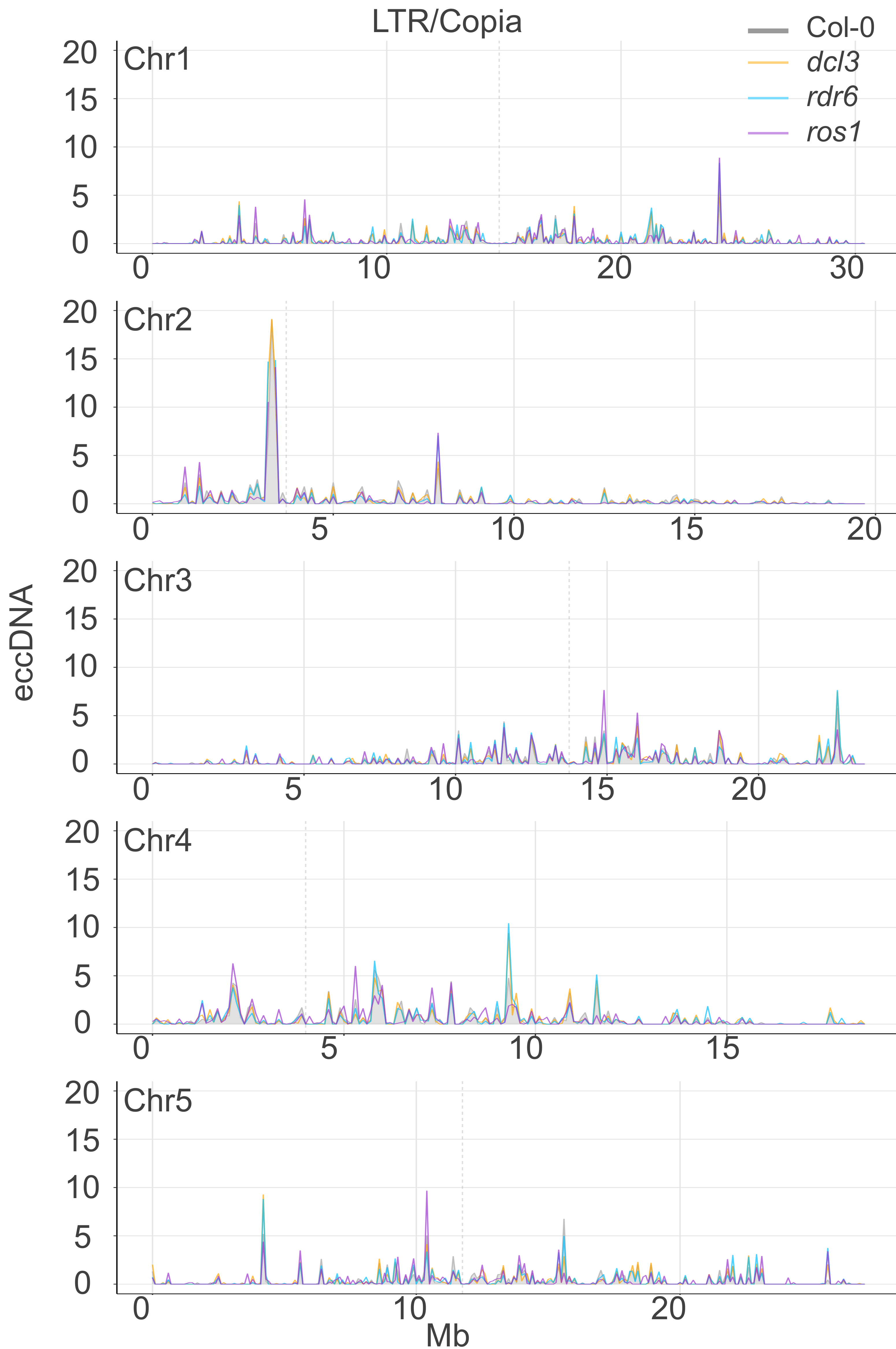

Supplement: S10 Fig — Col-0, dcl3, rdr6, and ros1 leaf tissues were processed on the CIDER-Seq pipeline (three replicates for each plant type). LTR/Copia-derived eccDNA reads are depicted on the Arabidopsis genome; the y-axis in each panel indicates the normalized eccDNA reads mapped per 100 kb bins on Arabidopsis chromosomes. (TIFF) [file pbio.3003275.s010.tiff]

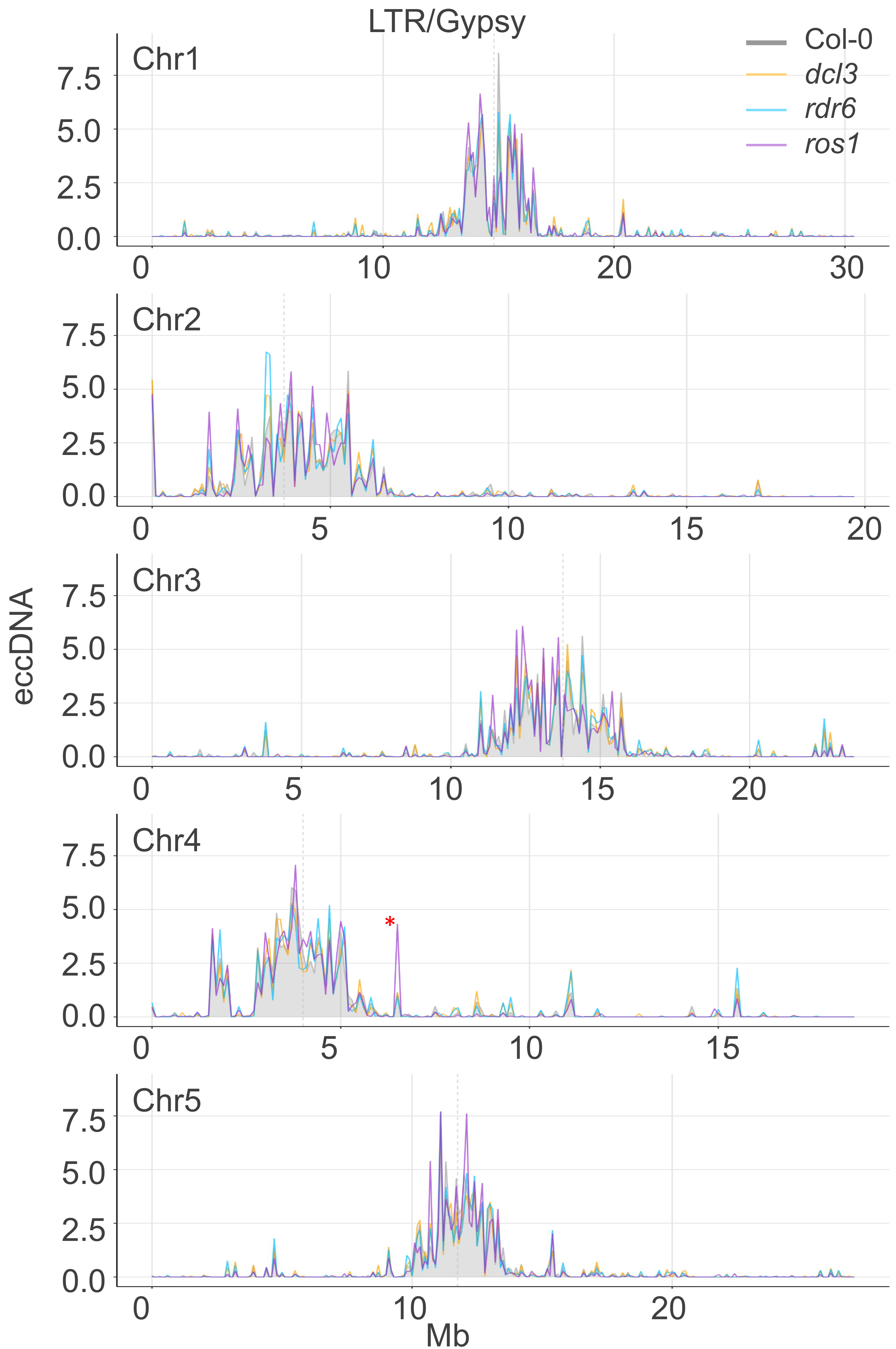

Supplement: S11 Fig — Col-0, dcl3, rdr6, and ros1 leaf tissues were processed on the CIDER-Seq pipeline (three replicates for each plant type). LTR/Gypsy-derived eccDNA reads are depicted on the Arabidopsis genome; the y-axis in each panel indicates the normalized eccDNA reads mapped per 100 kb bins on Arabidopsis chromosomes. The genomic region on Chr4, represented in Fig 3b, has been highlighted with a red asterisk. (TIFF) [file pbio.3003275.s011.tiff]

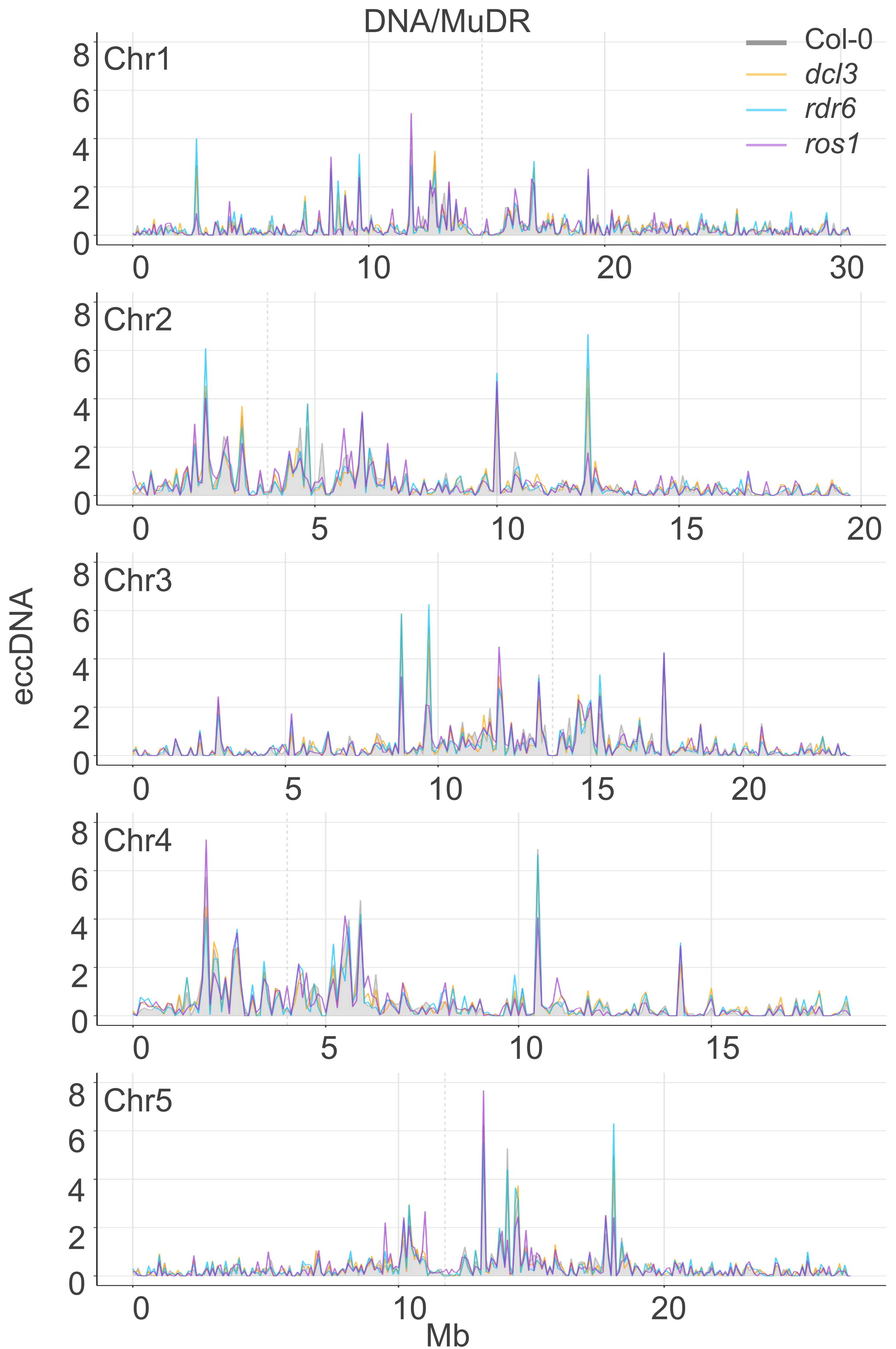

Supplement: S12 Fig — Col-0, dcl3, rdr6, and ros1 leaf tissues were processed on the CIDER-Seq pipeline (three replicates for each plant type). DNA/MuDR-derived eccDNA reads are depicted on the Arabidopsis genome; the y-axis in each panel indicates the normalized eccDNA reads mapped per 100 kb bins on Arabidopsis chromosomes. (TIFF) [file pbio.3003275.s012.tiff]

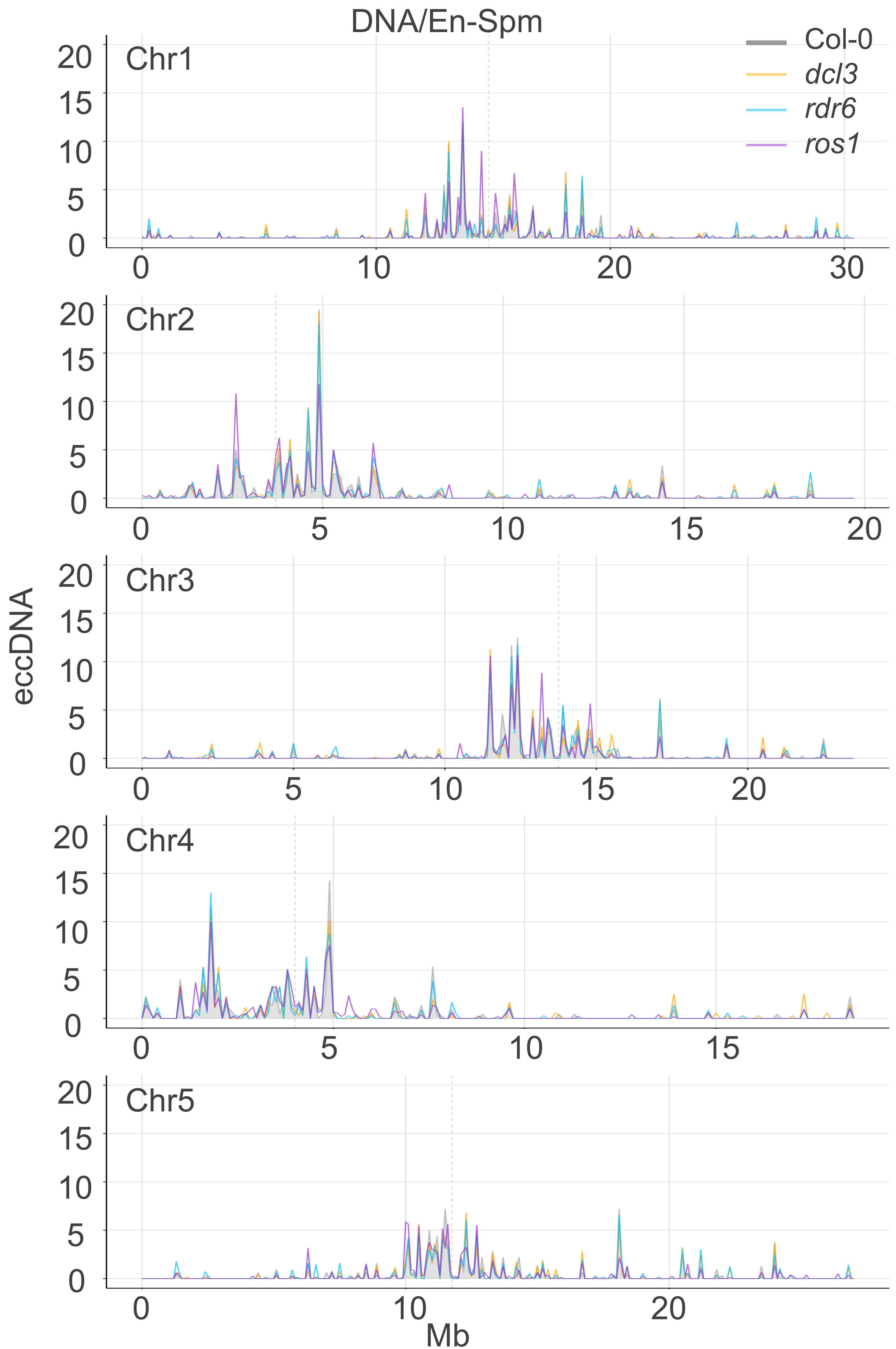

Supplement: S13 Fig — Col-0, dcl3, rdr6, and ros1 leaf tissues were processed on the CIDER-Seq pipeline (three replicates for each plant type). DNA/En-Spm-derived eccDNA reads are depicted on the Arabidopsis genome; the y-axis in each panel indicates the normalized eccDNA reads mapped per 100 kb bins on Arabidopsis chromosomes. (TIFF) [file pbio.3003275.s013.tiff]

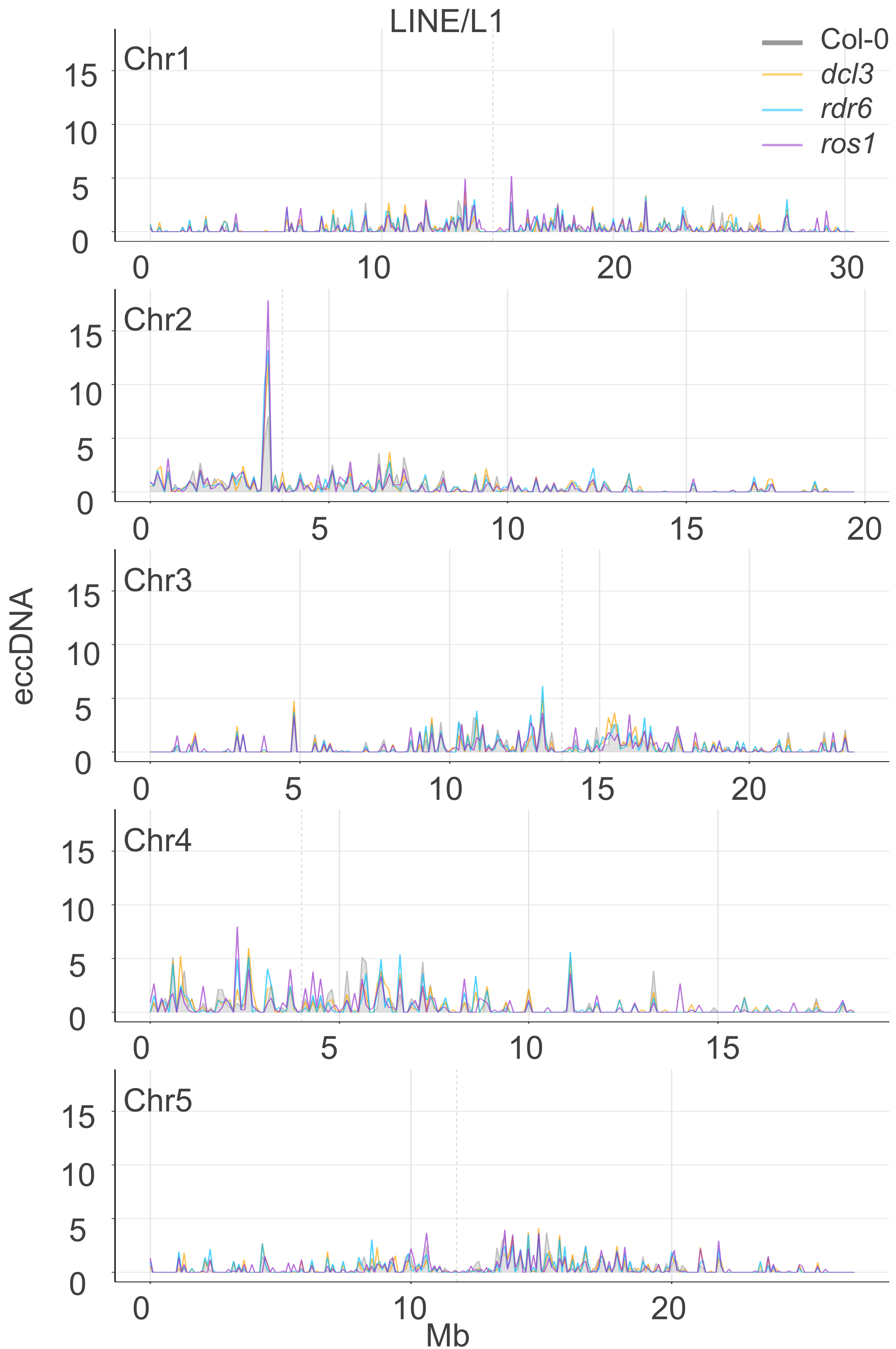

Supplement: S14 Fig — Col-0, dcl3, rdr6, and ros1 leaf tissues were processed on the CIDER-Seq pipeline (three replicates for each plant type). LINE/L1-derived eccDNA reads are depicted on the Arabidopsis genome; the y-axis in each panel indicates the normalized eccDNA reads mapped per 100 kb bins on Arabidopsis chromosomes. (TIFF) [file pbio.3003275.s014.tiff]

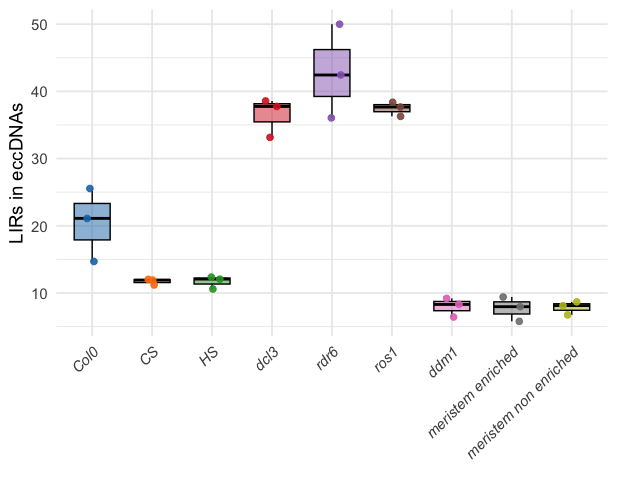

Supplement: S15 Fig — The eccDNAs are expressed as % of total LIR-derived eccDNAs along y-axis, across different genetic backgrounds and experimental conditions (x-axis). Each dot represents an individual biological replicate (n = 3 per condition). EccDNAs corresponding to LIRs were enriched in methylation mutants (dcl3, rdr6, ros1) but not in ddm1. The raw data supporting all figures can be found in S1 Data. (TIFF) [file pbio.3003275.s015.tiff]

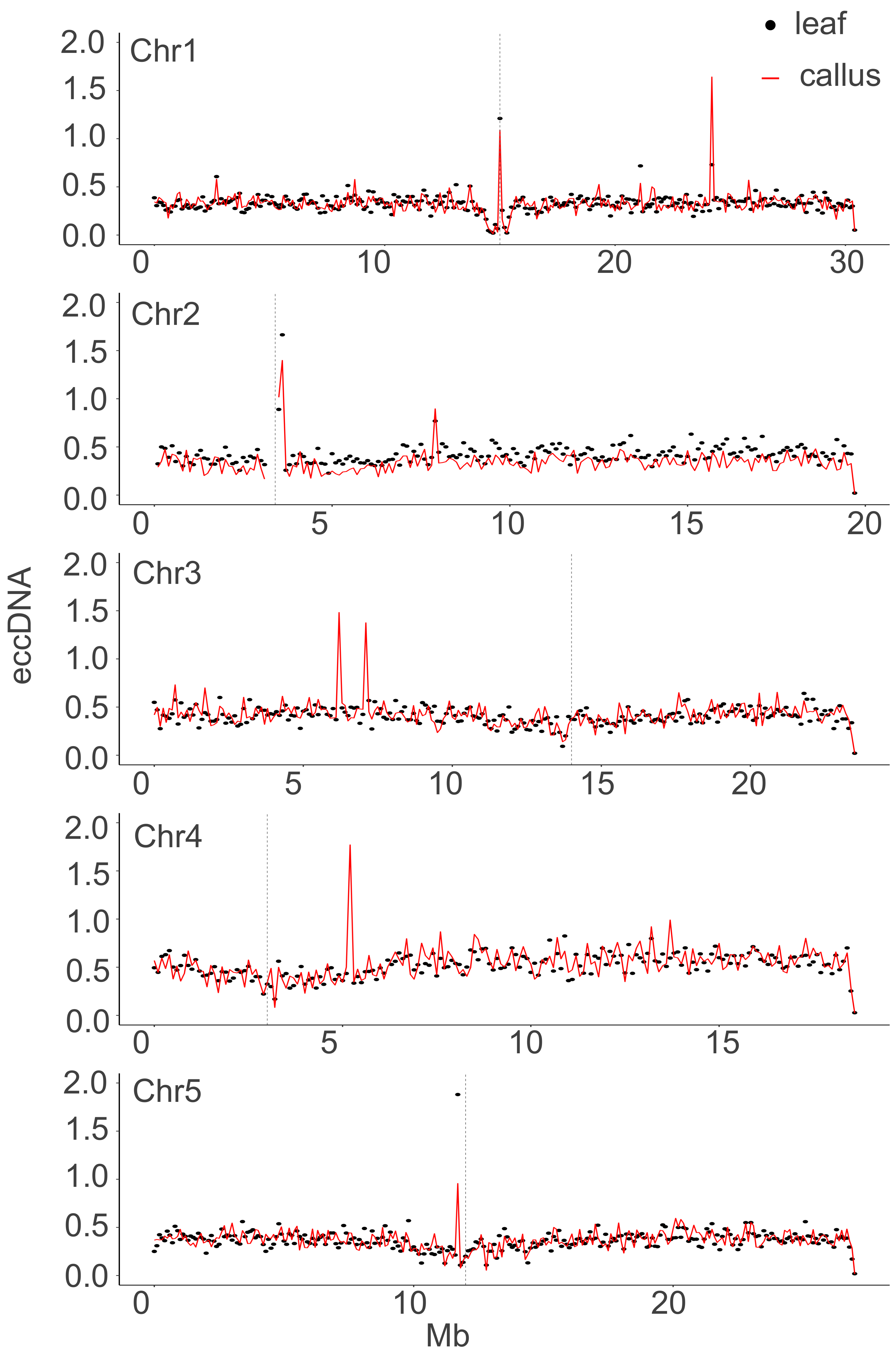

Supplement: S16 Fig — DNA extracted from calli induced from the Col-0 leaves was processed on the CIDER-Seq pipeline (three replicates for each condition). eccDNA reads from callus and leaf were mapped on the Arabidopsis genome; the y-axis in each panel indicates the normalized eccDNA reads mapped per 100 kb bins on Arabidopsis chromosomes. The raw data supporting all figures can be found in S1 Data. (TIFF) [file pbio.3003275.s016.tiff]

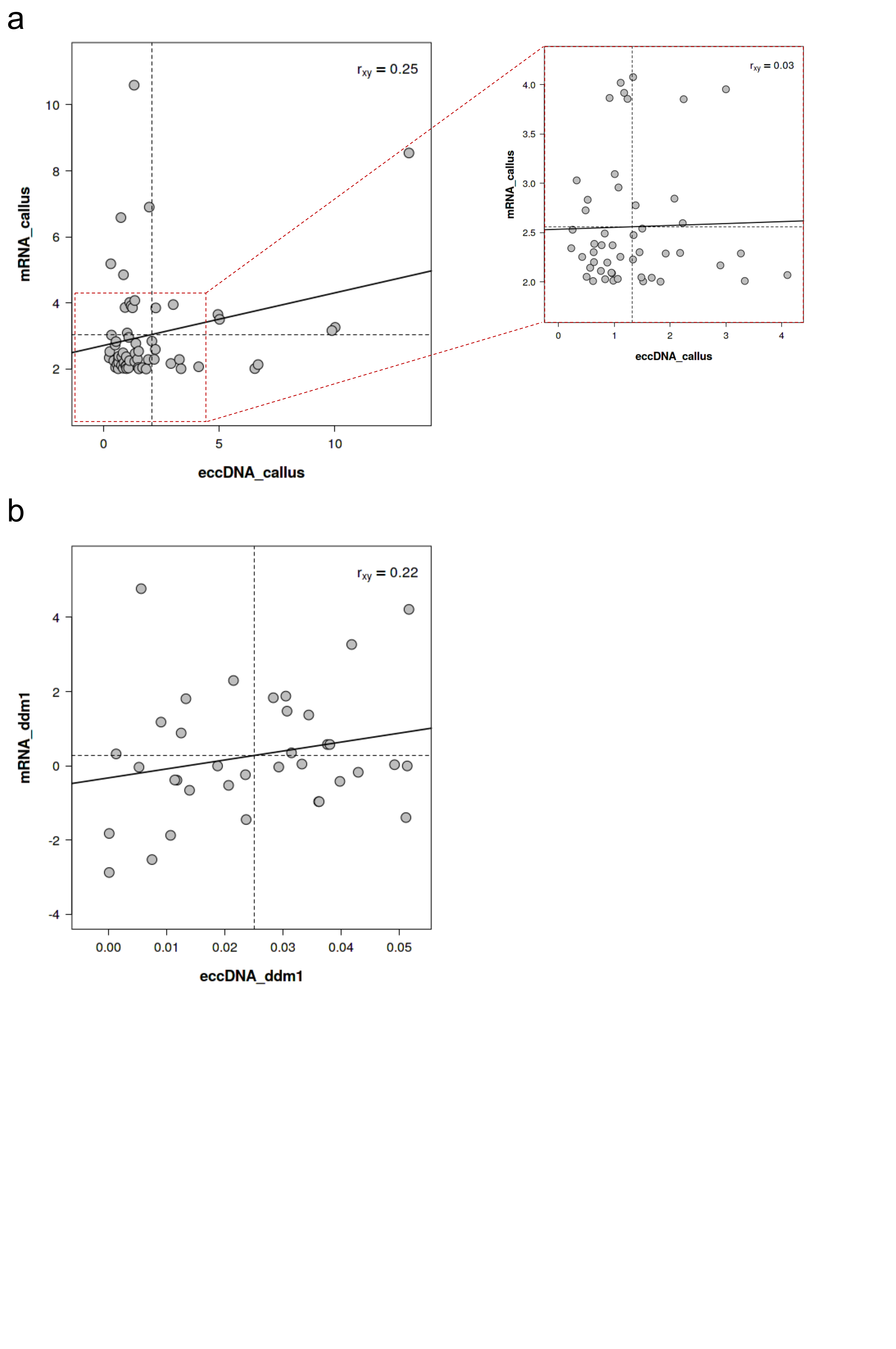

Supplement: S17 Fig — (a) Correlation analysis between transcript fold change calli/leaf and eccDNA fold change calli/leaf. The right panel indicates the same analysis on the clustered area. (b) Correlation analysis between transcript fold change meristem/non-meristem cells and eccDNA fold change meristem/non-meristem cells in Arabidopsis ddm1. The raw data supporting all figures can be found in S1 Data. (TIFF) [file pbio.3003275.s017.tiff]

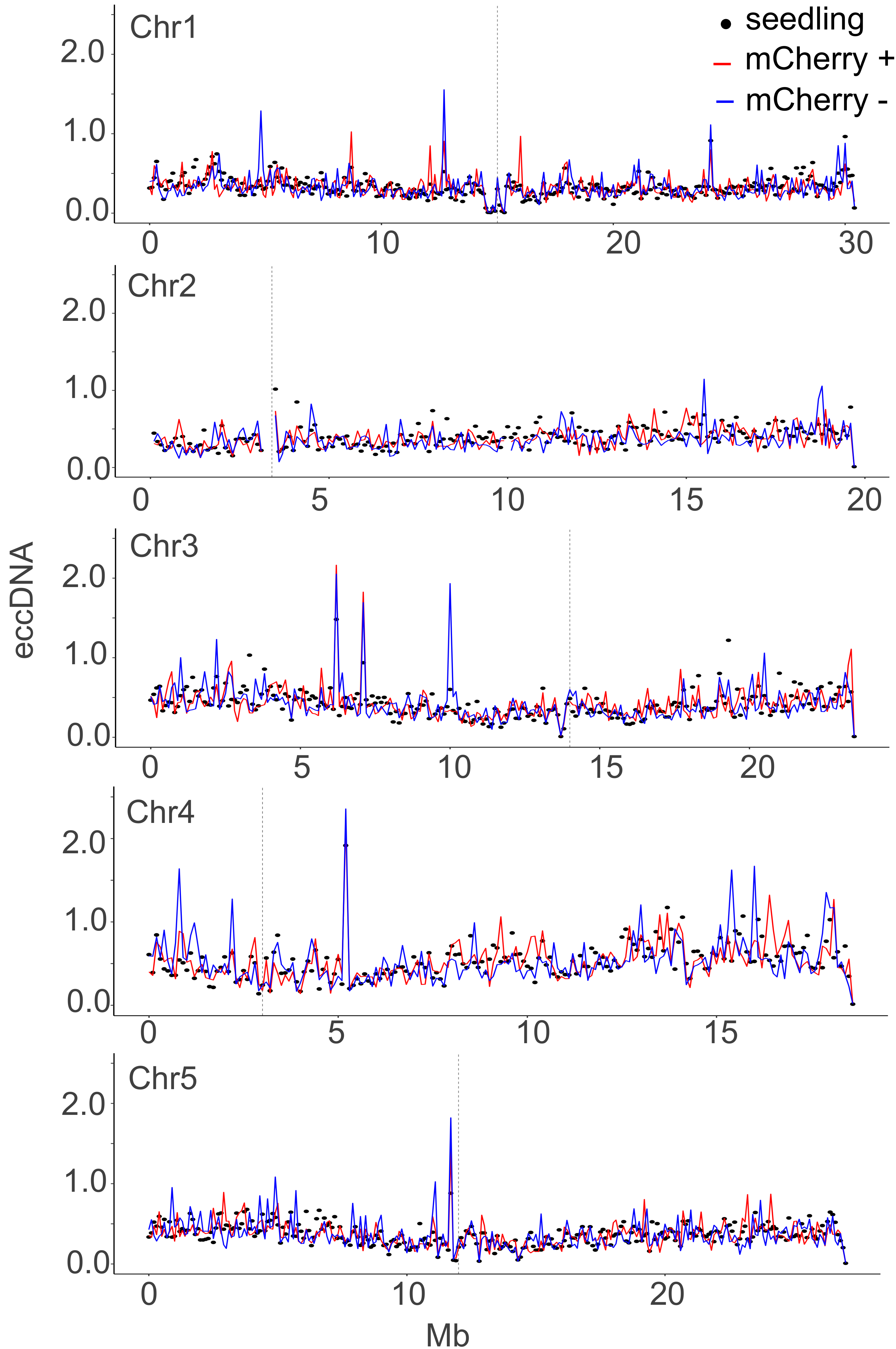

Supplement: S18 Fig — Shoot apical meristem (SAM) cells were sorted using fluorescence-activated nuclear sorting (FANS) and processed, with respective controls from ddm1 seedlings, on the CIDER-Seq pipeline for eccDNA amplification (three replicates for each cell type). EccDNA reads from SAM (mCherry +), surrounding cells (mCherry −), and ddm1 seedlings were mapped on the Arabidopsis genome; the y-axis in each panel indicates the normalized eccDNA reads mapped per 100 kb bins on Arabidopsis chromosomes. The raw data supporting all figures can be found in S1 Data. (TIFF) [file pbio.3003275.s018.tiff]

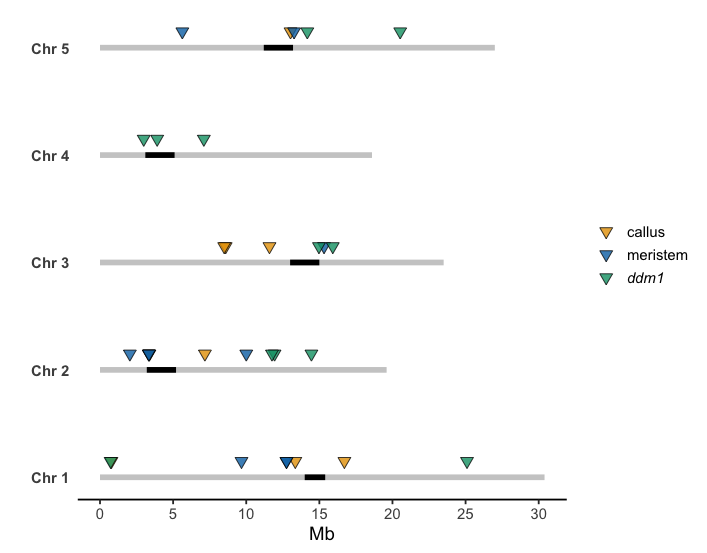

Supplement: S19 Fig — Fig 3. Schematic representation of Arabidopsis chromosomes 1–5. Grey bars represent chromosomes, with black segments indicating centromeres. Arrowheads indicate the genomic positions of TEs shown in Fig 3, with colors corresponding to the sample in which their eccDNAs were detected: callus (orange), meristem (blue), and ddm1 (green). Chromosome coordinates are shown in megabases (Mb). (TIFF) [file pbio.3003275.s019.tiff]

Figure S2C

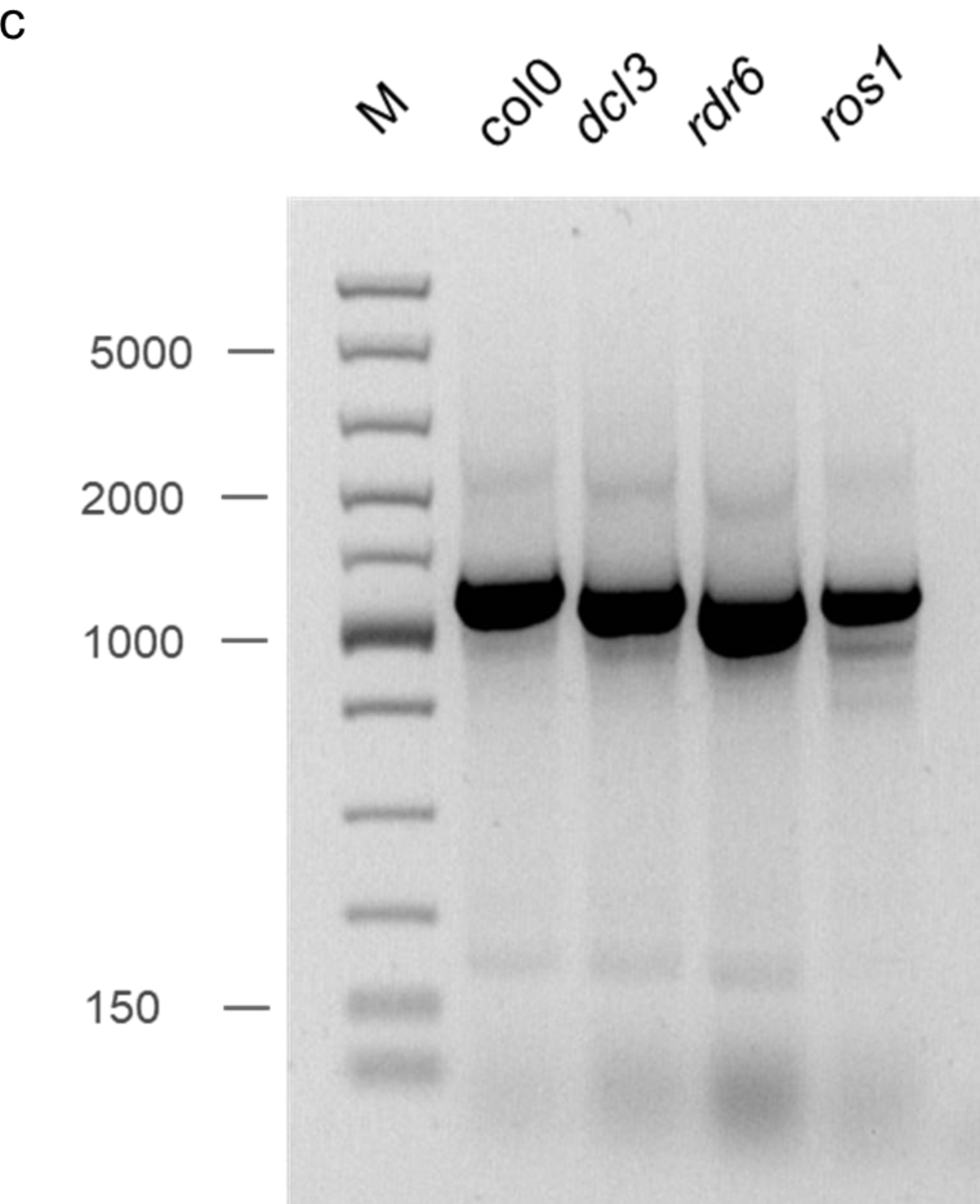

Raw images

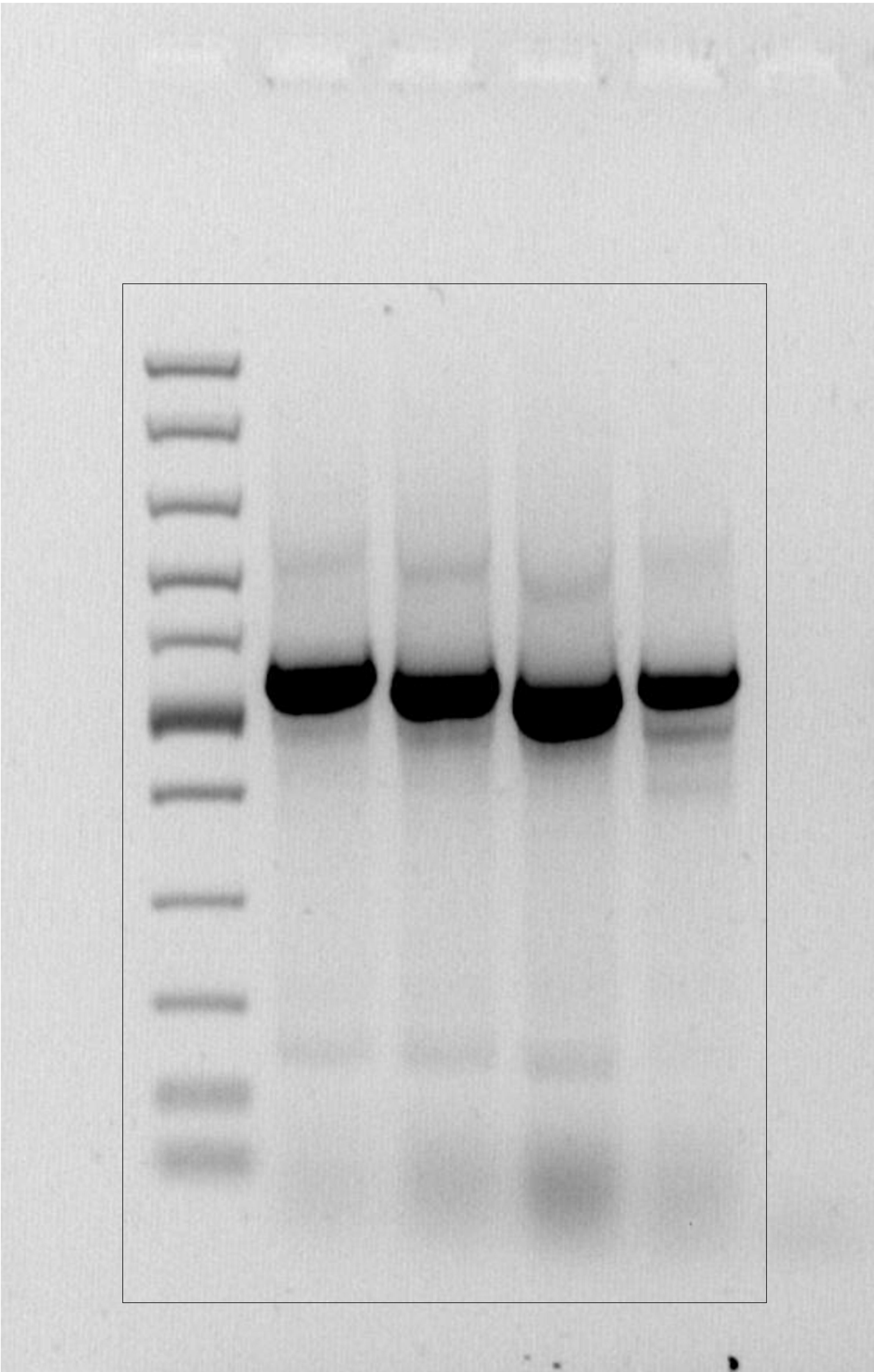

Figure S4

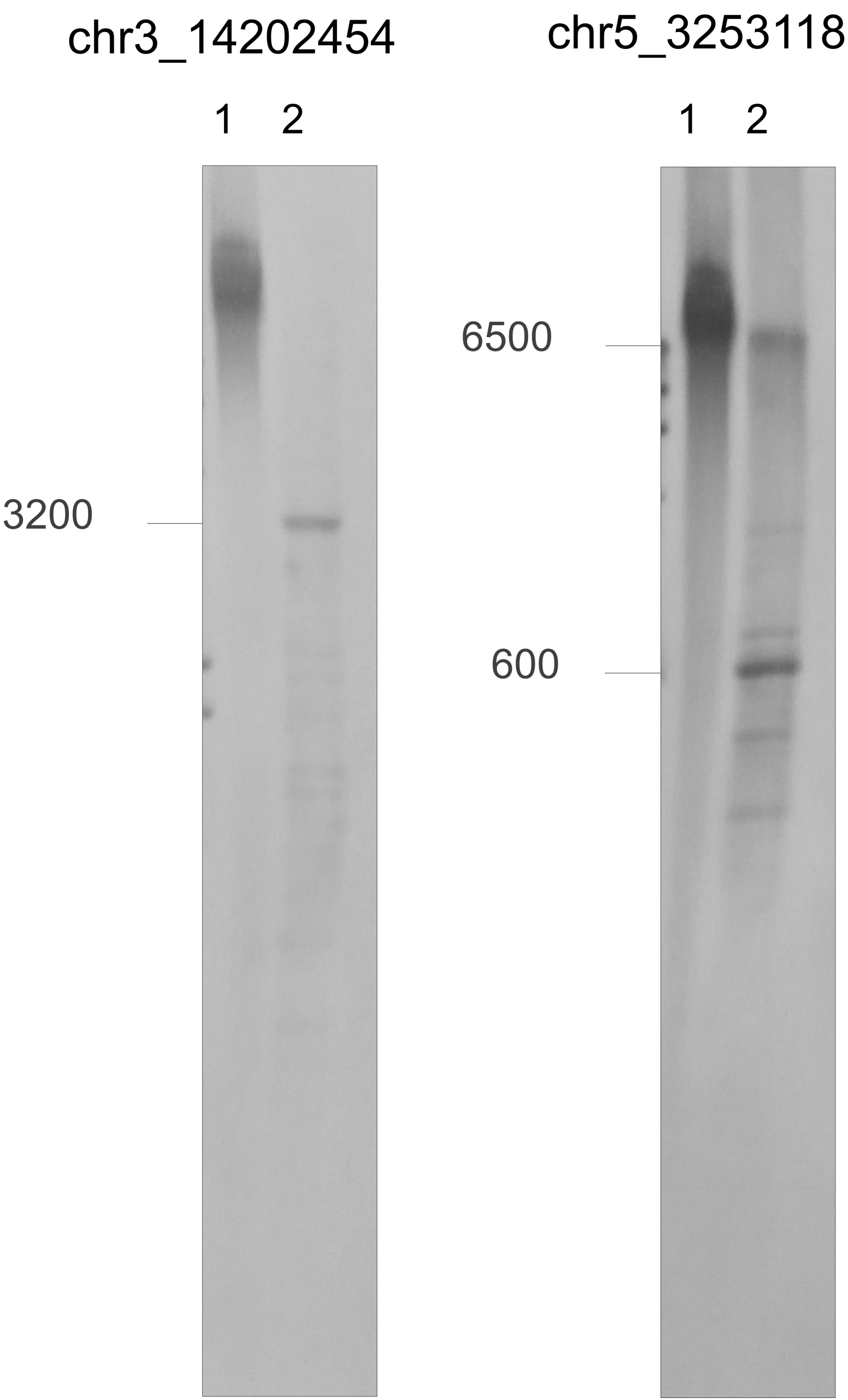

Raw images

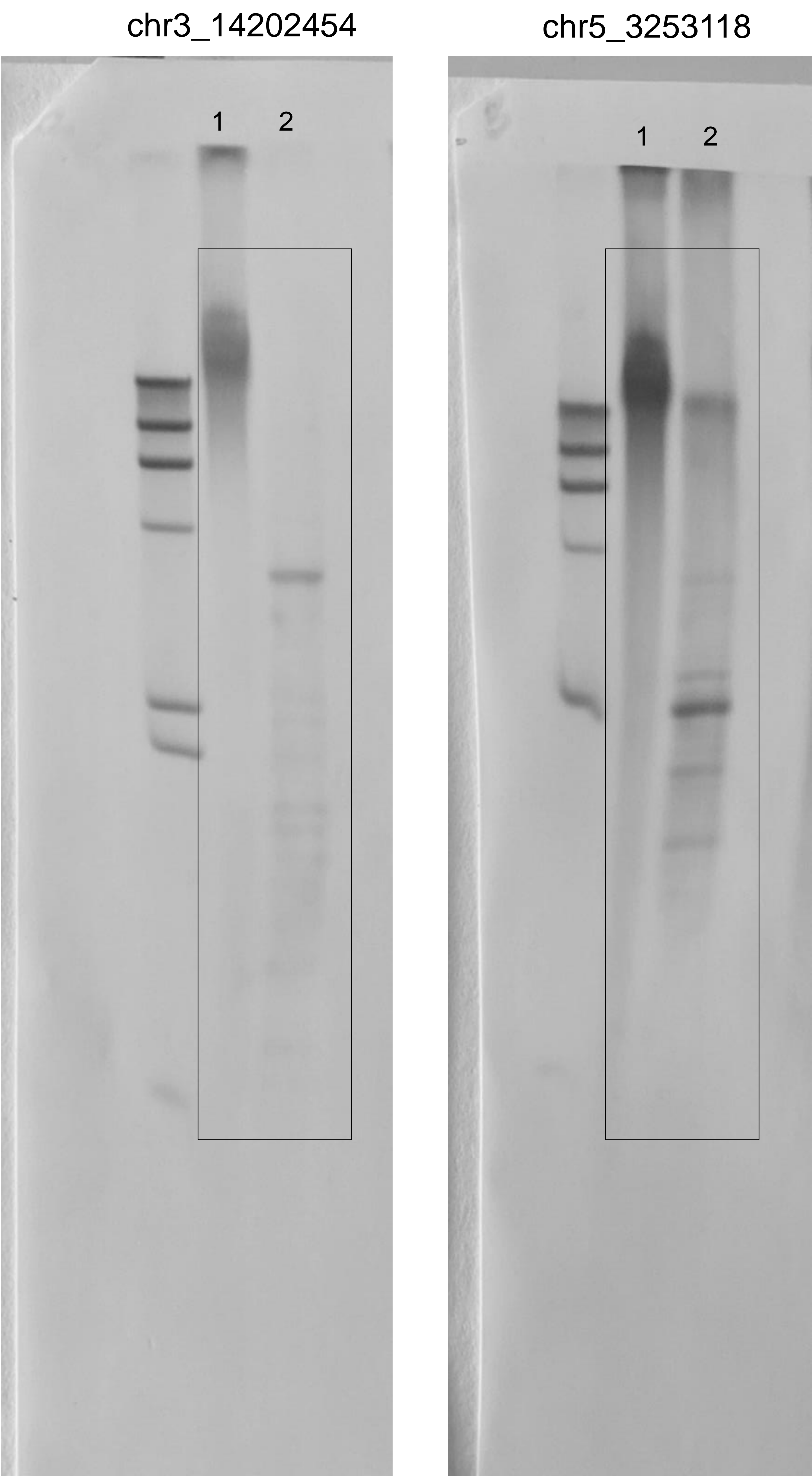

Supplement: S1 Raw Images — (PDF) [file pbio.3003275.s031.pdf]
