# Supplementary material for: A comprehensive atlas of full-length Arabidopsis eccDNA populations identifies their genomic origins and epigenetic regulation
Source: PLoS Biol. 2025 Jul 15;23(7):e3003275. doi: 10.1371/journal.pbio.3003275 (PMC12273906; doi:10.1371/journal.pbio.3003275)
Supplement: S1 Table — Demultiplexed data from Sequel II is presented in Mb with the number of reads in corresponding samples; demultiplexing is performed with LIMA. Clustering is performed with cd-hit where unique clusters represent the clusters with single reads. CCS = circular consensus sequencing, NS = Col-0 non-stressed, CS = control stress, HS = heat stress. (DOCX) [file pbio.3003275.s020.docx]

## S1 Table. PacBio sequencing of eccDNA; data, read statistics, and clustering.

Demultiplexed data from Sequel II is presented in Mb with the number of reads in corresponding samples; demultiplexing is performed with LIMA. Clustering is performed with cd-hit where unique clusters represent the clusters with single reads. CCS = circular consensus sequencing, NS = Col-0 non-stressed, CS = control stress, HS = heat stress.

| **Sample** | **Condition** | **CCS reads** | **eccDNA** | | | **Clustering** | |
| --- | --- | --- | --- | --- | --- | --- | --- |
|  |  | **No.** | **No.** | **bases** | **Mean length** | **No. of clusters** | **Unique clusters** |
| cs1 | control stress-1 | 11727 | 4451 | 1.4E+07 | 3165.7 | 3845 | 3555 |
| cs2 | control stress-2 | 12482 | 4765 | 1.6E+07 | 3297 | 3932 | 3553 |
| cs3 | control stress-3 | 14569 | 4956 | 1.8E+07 | 3599.7 | 4011 | 3670 |
| hs1 | heat stress-1 | 15906 | 6445 | 2.1E+07 | 3184.6 | 5164 | 4634 |
| hs2 | heat stress-2 | 9180 | 4067 | 1.4E+07 | 3437 | 3506 | 3226 |
| hs3 | heat stress-3 | 9984 | 3206 | 1.1E+07 | 3360 | 2774 | 2580 |
| ns1 | No stress Col0-1 | 32887 | 398 | 1672072 | 4201.2 | 297 | 270 |
| ns2 | No stress Col0-2 | 111946 | 3376 | 5003134 | 1482 | 2311 | 2151 |
| ns3 | No stress Col0-3 | 18967 | 415 | 1471814 | 3546.5 | 274 | 235 |
| plus5k-1 | mCherry + 1 | 43525 | 942 | 3057522 | 3245.8 | 757 | 37 |
| plus5k-2 | mCherry + 2 | 35391 | 638 | 2260398 | 3309.5 | 515 | 36 |
| plus5k-3 | mCherry + 3 | 32121 | 794 | 2224509 | 2801.6 | 484 | 451 |
| min10k1 | mCherry - 1 | 98615 | 2282 | 6970826 | 3054.7 | 1639 | 1505 |
| min10k2 | mCherry - 2 | 53996 | 1441 | 4584957 | 3181.8 | 879 | 816 |
| min10k3 | mCherry - 3 | 32568 | 593 | 2044158 | 3447.1 | 310 | 277 |
| ddm1 -1 | *ddm1* -1 | 78437 | 1555 | 5217786 | 3355.5 | 1287 | 1197 |
| ddm1 -2 | *ddm1* -2 | 48134 | 936 | 3027933 | 3235 | 826 | 789 |
| ddm1 -3 | *ddm1* -3 | 58508 | 1725 | 4624944 | 2681.1 | 1243 | 1133 |
| cal1 | callus-1 | 25674 | 426 | 1640948 | 3852 | 332 | 302 |
| cal2 | callus-2 | 50259 | 844 | 3248366 | 3848.8 | 536 | 472 |
| cal3 | callus-3 | 33212 | 674 | 2708945 | 3474.3 | 442 | 403 |
| ros1 -1 | *ros1* -1 | 68186 | 804 | 2814933 | 3501.2 | 441 | 394 |
| ros1 -2 | *ros1* -2 | 81264 | 1082 | 4528322 | 4185.1 | 757 | 668 |
| ros1 -3 | *ros1* -3 | 74668 | 827 | 3198883 | 3868.1 | 487 | 417 |
| rdr6 -1 | *rdr6* -1 | 70251 | 807 | 3718641 | 4608 | 556 | 468 |
| rdr6 -2 | *rdr6* -2 | 63685 | 781 | 3290653 | 4213.4 | 484 | 421 |
| rdr6 -3 | *rdr6* -3 | 84428 | 1244 | 6921003 | 5563.5 | 932 | 824 |
| dcl3 -1 | *dcl3* -1 | 65855 | 847 | 4638491 | 5476.4 | 619 | 542 |
| dcl3 -2 | *dcl3* -2 | 78366 | 1136 | 6199007 | 5456.9 | 869 | 755 |
| dcl3 -3 | *dcl3* -3 | 63497 | 1307 | 6807898 | 5208.8 | 767 | 670 |

* CCS was performed with 0.99 % accuracy

** Based on >1 round of deconcat
