# Supplementary material for: A comprehensive atlas of full-length Arabidopsis eccDNA populations identifies their genomic origins and epigenetic regulation
Source: PLoS Biol. 2025 Jul 15;23(7):e3003275. doi: 10.1371/journal.pbio.3003275 (PMC12273906; doi:10.1371/journal.pbio.3003275)
Supplement: S2 Table — (DOCX) [file pbio.3003275.s021.docx]

## S2 Table: Primers used in this study

| **Primer** | **Sequence** |
| --- | --- |
| ONSEN_F1 | TAATGTTCCCTTCCAAGTCCC |
| ONSEN_R1 | GCTTGTAATGACCCAAGAAGT |
| ONSEN_F2 | AAGTCGGCAATAGCTTTGGCGAAGA |
| chr3_14202454-F | CGAAGGTGCATAGTGAGAAGAGTAA |
| chr3_14202454-R | GTTCCCTTGGCTGTGGTTTCGCTG |
| chr5_3253118-F | GGTAATATCATGCGAACAAG |
| chr5_3253118-R | GTGGCGGTATAGTCGTCTTG |
| chr2_3416272-F | TGGCATTTGAGTTGTCTCCC |
| chr2_3416272-R | CCAATATGGATGGCTTGCCT |
| chr2_3436423 | ACCTTTTGAGTCACAGCCAC |
| chr2_3436423 | TGAAGAATCTCCTGTTGCGT |
