# Supplementary material for: A comprehensive atlas of full-length Arabidopsis eccDNA populations identifies their genomic origins and epigenetic regulation
Source: PLoS Biol. 2025 Jul 15;23(7):e3003275. doi: 10.1371/journal.pbio.3003275 (PMC12273906; doi:10.1371/journal.pbio.3003275)
Supplement: S3 Table — (DOCX) [file pbio.3003275.s022.docx]

## S3 Table: Gene ontology of eccDNA

| **GO ID** | **Arabidopsis thaliana - REFLIST (27436)** | **upload_1 (338)** | **upload_1 (expected)** | **upload_1 (over/under)** | **upload_1 (fold Enrichment)** | **upload_1 (P-value)** |
| --- | --- | --- | --- | --- | --- | --- |
| RNA modification (GO:0009451) | 362 | 25 | 4.46 | + | 5.61 | 1.29E-08 |
| mRNA metabolic process (GO:0016071) | 483 | 20 | 5.95 | + | 3.36 | 7.87E-03 |
| RNA metabolic process (GO:0016070) | 1455 | 48 | 17.92 | + | 2.68 | 1.45E-06 |
| nucleic acid metabolic process (GO:0090304) | 1907 | 57 | 23.49 | + | 2.43 | 1.24E-06 |
| nucleobase-containing compound metabolic process (GO:0006139) | 2323 | 62 | 28.62 | + | 2.17 | 1.62E-05 |
| heterocycle metabolic process (GO:0046483) | 2660 | 64 | 32.77 | + | 1.95 | 6.25E-04 |
| cellular aromatic compound metabolic process (GO:0006725) | 2755 | 64 | 33.94 | + | 1.89 | 1.73E-03 |
| organic cyclic compound metabolic process (GO:1901360) | 2875 | 66 | 35.42 | + | 1.86 | 1.75E-03 |
| cellular nitrogen compound metabolic process (GO:0034641) | 3039 | 68 | 37.44 | + | 1.82 | 3.24E-03 |
| Unclassified (UNCLASSIFIED) | 5680 | 65 | 69.98 | - | 0.93 | 0.00E+00 |
