# Supplementary material for: A comprehensive atlas of full-length Arabidopsis eccDNA populations identifies their genomic origins and epigenetic regulation
Source: PLoS Biol. 2025 Jul 15;23(7):e3003275. doi: 10.1371/journal.pbio.3003275 (PMC12273906; doi:10.1371/journal.pbio.3003275)
Supplement: S5 Table — (DOCX) [file pbio.3003275.s024.docx]

## S5 Table. eccDNA characterization and the number of full-length TEs in eccDNAs.

| **Sample** | **Condition** | **CCS reads** | **eccDNA** | **TE eccDNA** | **Full TE in eccDNA** | |
| --- | --- | --- | --- | --- | --- | --- |
|  |  | **No.** | **No.** | **No.** | **No.** | **percent** |
| cs1 | control stress-1 | 11727 | 4451 | 1659 | 861 | 51.898 |
| cs2 | control stress-2 | 12482 | 4765 | 1691 | 884 | 52.276 |
| cs3 | control stress-3 | 14569 | 4956 | 1869 | 1006 | 53.825 |
| hs1 | heat stress-1 | 15906 | 6445 | 2267 | 1236 | 54.521 |
| hs2 | heat stress-2 | 9180 | 4067 | 1579 | 832 | 52.691 |
| hs3 | heat stress-3 | 9984 | 3206 | 1178 | 641 | 54.414 |
| wt1 | No stress Col0-1 | 32887 | 398 | 141 | 88 | 62.411 |
| wt2 | No stress Col0-2 | 111946 | 3376 | 688 | 231 | 33.575 |
| wt3 | No stress Col0-3 | 18967 | 415 | 112 | 65 | 58.035 |
| plus5k-1 | mCherry + 1 | 43525 | 942 | 291 | 140 | 48.11 |
| plus5k-2 | mCherry + 2 | 35391 | 638 | 211 | 102 | 48.341 |
| plus5k-3 | mCherry + 3 | 32121 | 794 | 204 | 92 | 45.098 |
| min10k1 | mCherry - 1 | 98615 | 2282 | 669 | 305 | 45.590 |
| min10k2 | mCherry - 2 | 53996 | 1441 | 422 | 185 | 43.838 |
| min10k3 | mCherry - 3 | 32568 | 593 | 160 | 77 | 48.125 |
| ddm1 -1 | *ddm1* -1 | 78437 | 1555 | 487 | 267 | 54.825 |
| ddm1 -2 | *ddm1* -2 | 48134 | 936 | 326 | 161 | 49.386 |
| ddm1 -3 | *ddm1* -3 | 58508 | 1725 | 442 | 202 | 45.701 |
| cal1 | callus-1 | 25674 | 426 | 153 | 85 | 55.555 |
| cal2 | callus-2 | 50259 | 844 | 258 | 139 | 53.876 |
| cal3 | callus-3 | 47519 | 585 | 166 | 83 | 52.970 |
| ros1 -1 | *ros1* -1 | 68186 | 804 | 198 | 110 | 55.555 |
| ros1 -2 | *ros1* -2 | 81264 | 1082 | 339 | 185 | 54.572 |
| ros1 -3 | *ros1* -3 | 74668 | 827 | 231 | 118 | 51.082 |
| rdr6 -1 | *rdr6* -1 | 70251 | 807 | 271 | 171 | 63.099 |
| rdr6 -2 | *rdr6* -2 | 63685 | 781 | 220 | 122 | 55.454 |
| rdr6 -3 | *rdr6* -3 | 84428 | 1244 | 527 | 338 | 64.136 |
| dcl3 -1 | *dcl3* -1 | 65855 | 847 | 339 | 203 | 59.882 |
| dcl3 -2 | *dcl3* -2 | 78366 | 1136 | 516 | 311 | 60.271 |
| dcl3 -3 | *dcl3* -3 | 63497 | 1307 | 386 | 250 | 64.766 |
