# Supplementary material for: A comprehensive atlas of full-length Arabidopsis eccDNA populations identifies their genomic origins and epigenetic regulation
Source: PLoS Biol. 2025 Jul 15;23(7):e3003275. doi: 10.1371/journal.pbio.3003275 (PMC12273906; doi:10.1371/journal.pbio.3003275)
Supplement: S6 Table — (DOCX) [file pbio.3003275.s025.docx]

## S6 Table: TE-derived eccDNAs from respective TE superfamilies in *Arabidopsis* col-0 and mutants.

| **TE superfamily / Plant** | **Col-0 1** | **Col-0 2** | **Col-0 3** | **dcl3 1** | **dcl3 2** | **dcl3 3** | **rdr6 1** | **rdr6 2** | **rdr6 3** | **ros1 1** | **ros1 2** | **ros1 3** | **ddm1 1** | **ddm1 2** | **ddm1 3** |
| --- | --- | --- | --- | --- | --- | --- | --- | --- | --- | --- | --- | --- | --- | --- | --- |
| **RC/Helitron** | 60 | 266 | 61 | 142 | 219 | 159 | 105 | 86 | 233 | 94 | 132 | 102 | 183 | 118 | 167 |
| **LTR/Gypsy** | 20 | 117 | 13 | 72 | 98 | 70 | 58 | 57 | 81 | 39 | 71 | 47 | 77 | 43 | 70 |
| **DNA/MuDR** | 22 | 127 | 12 | 49 | 69 | 56 | 37 | 32 | 88 | 33 | 48 | 29 | 83 | 68 | 79 |
| **LTR/Copia** | 13 | 56 | 7 | 29 | 39 | 41 | 18 | 16 | 45 | 12 | 34 | 22 | 47 | 24 | 33 |
| **DNA** | 11 | 30 | 7 | 13 | 26 | 28 | 10 | 8 | 22 | 12 | 13 | 13 | 24 | 13 | 15 |
| **LINE/L1** | 4 | 29 | 6 | 13 | 14 | 22 | 12 | 6 | 28 | 7 | 17 | 9 | 20 | 16 | 27 |
| **DNA/En-Spm** | 6 | 31 | 5 | 14 | 25 | 12 | 14 | 10 | 18 | 7 | 11 | 18 | 20 | 18 | 18 |
| **DNA/HAT** | 4 | 12 | 5 | 8 | 21 | 10 | 5 | 6 | 13 | 3 | 7 | 6 | 9 | 6 | 12 |
| **Unassigned** | 1 | 9 | 2 | 2 | 3 | 3 | 7 | 2 | 10 | 5 | 6 | 1 | 6 | 5 | 5 |
| **DNA/Harbinger** | 1 | 4 | 1 | 3 | 5 | 5 | 5 | 4 | 5 | 2 | 3 | 1 | 7 | 7 | 6 |
| **DNA/Pogo** | 2 | 11 |  | 2 | 5 | 2 | 1 | 1 | 5 |  | 4 | 3 | 3 | 4 | 4 |
| **LINE?** |  | 5 |  | 1 | 2 |  |  |  | 1 |  | 4 | 1 | 1 | 1 | 1 |
| **DNA/Tc1** |  | 1 |  |  | 2 | 1 |  | 1 | 3 |  |  | 2 | 2 | 2 | 1 |
| **RathE1_cons** |  |  |  | 1 | 1 | 1 | 1 |  | 2 |  |  | 1 | 3 |  | 2 |
| **DNA/Mariner** | 1 | 1 | 1 | 1 | 2 | 1 | 1 |  | 1 |  |  | 1 |  |  |  |
| **RathE3_cons** |  | 2 |  |  | 1 | 2 |  |  |  |  | 1 |  | 1 |  | 2 |
| **RathE2_cons** | 1 | 2 |  |  | 1 |  |  | 1 |  |  |  |  |  | 1 |  |
| **SINE** |  | 2 |  |  |  |  | 1 |  | 1 |  |  |  | 1 |  |  |
