# Supplementary material for: A comprehensive atlas of full-length Arabidopsis eccDNA populations identifies their genomic origins and epigenetic regulation
Source: PLoS Biol. 2025 Jul 15;23(7):e3003275. doi: 10.1371/journal.pbio.3003275 (PMC12273906; doi:10.1371/journal.pbio.3003275)
Supplement: S7 Table — (DOCX) [file pbio.3003275.s026.docx]

## S7 Table: TEs with most abundant eccDNA reads

| **TAIR id** | **TE copy No.** | | | | | | | | | **TE family** | **TE Super family** |
| --- | --- | --- | --- | --- | --- | --- | --- | --- | --- | --- | --- |
|  | **CS1** | **CS2** | **CS3** | **HS1** | **HS2** | **HS3** | **WT1** | **WT2** | **WT3** |  |  |
| AT4TE14890 | 31 | 43 | 80 | 83 | 41 | 29 | 48 | 74 | 41 | DNA | ATREP18 |
| AT2TE26350 | 10 | 12 | 26 | 15 | 9 | 5 | 20 | 28 | 24 | DNA | ATREP19 |
| AT1TE38090 | 19 | 22 | 50 | 59 | 23 | 19 | 41 | 48 | 49 | DNA/MuDR | VANDAL2N1 |
| AT2TE35755 | 26 | 27 | 15 | 35 | 11 | 6 | 17 | 22 | 26 | LTR/Copia | ATCOPIA28 |
| AT2TE16235 | 21 | 29 | 19 | 39 | 8 | 18 | 19 | 29 | 19 | LTR/Gypsy | ATLANTYS3 |
| AT4TE76870 | 17 | 30 | 27 | 29 | 19 | 11 | 16 | 30 | 30 | RC/Helitron | ATREP10 |
| AT2TE44930 | 13 | 21 | 37 | 23 | 11 | 15 | 26 | 39 | 27 | RC/Helitron | ATREP10B |
| AT5TE11315 | 32 | 20 | 38 | 39 | 16 | 12 | 49 | 28 | 25 | RC/Helitron | ATREP10D |
| AT1TE69930 | 13 | 14 | 14 | 20 | 3 | 11 | 27 | 26 | 25 | RC/Helitron | ATREP4 |
| AT3TE77985 | 6 | 10 | 21 | 27 | 9 | 8 | 17 | 21 | 15 | RC/Helitron | HELITRONY1B |
