# Supplementary material for: A comprehensive atlas of full-length Arabidopsis eccDNA populations identifies their genomic origins and epigenetic regulation
Source: PLoS Biol. 2025 Jul 15;23(7):e3003275. doi: 10.1371/journal.pbio.3003275 (PMC12273906; doi:10.1371/journal.pbio.3003275)
Supplement: S8 Table — (DOCX) [file pbio.3003275.s027.docx]

## S8 Table: TE-derived eccDNAs from respective TE families in *Arabidopsis* col-0 and mutants

| **TE family / Plant** | **Col-0 1** | **Col-0 2** | **Col-0 3** | **dcl3 1** | **dcl3 2** | **dcl3 3** | **rdr6 1** | **rdr6 2** | **rdr6 3** | **ros1 1** | **ros1 2** | **ros1 3** | **ddm1 1** | **ddm1 2** | **ddm1 3** |
| --- | --- | --- | --- | --- | --- | --- | --- | --- | --- | --- | --- | --- | --- | --- | --- |
| **ATREP3** | 5 | 32 | 11 | 22 | 22 | 11 | 20 | 15 | 27 | 6 | 20 | 13 | 23 | 18 | 20 |
| **ATREP4** | 5 | 31 | 7 | 14 | 22 | 14 | 7 | 7 | 25 | 19 | 11 | 13 | 11 | 17 | 16 |
| **ATREP10** | 8 | 33 | 4 | 17 | 18 | 19 | 7 | 9 | 18 | 11 | 8 | 5 | 26 | 7 | 26 |
| **HELITRONY3** | 7 | 22 | 4 | 10 | 20 | 12 | 7 | 9 | 16 | 9 | 19 | 8 | 15 | 5 | 7 |
| **ATREP10B** | 1 | 19 | 4 | 10 | 17 | 21 | 6 | 8 | 22 | 8 | 9 | 10 | 6 | 5 | 11 |
| **ATREP15** | 6 | 9 | 5 | 11 | 13 | 10 | 8 | 2 | 14 | 7 | 9 | 5 | 14 | 11 | 16 |
| **ATHILA2** | 4 | 13 | 1 | 11 | 14 | 12 | 9 | 12 | 11 | 6 | 8 | 2 | 6 | 11 | 6 |
| **ATREP11** | 4 | 19 | 3 | 10 | 11 | 9 | 7 | 4 | 9 | 6 | 6 | 4 | 10 | 9 | 11 |
| **ATREP19** | 5 | 6 | 6 | 6 | 12 | 14 | 3 | 4 | 10 | 6 | 5 | 7 | 8 | 3 | 8 |
| **HELITRONY1D** | 2 | 16 | 3 | 6 | 9 | 5 | 3 | 1 | 14 | 5 | 3 | 2 | 14 | 7 | 8 |
| **ATREP5** | 1 | 13 |  | 5 | 14 | 9 | 5 | 5 | 11 | 4 | 8 |  | 7 | 6 | 10 |
| **ATHILA6A** | 1 | 13 | 5 | 7 | 14 | 5 | 9 | 3 | 7 | 5 | 4 | 4 | 9 | 2 | 10 |
| **ATGP1** | 2 | 7 | 1 | 9 | 11 | 8 | 4 | 5 | 6 | 1 | 8 | 1 | 5 | 4 | 9 |
| **ATREP1** | 1 | 5 | 3 | 2 | 9 | 11 | 8 | 4 | 6 | 1 | 5 | 5 | 7 | 4 | 8 |
| **ATHILA3** | 1 | 7 | 1 | 4 | 9 | 7 | 5 | 7 | 11 | 1 | 4 | 3 | 3 | 2 | 6 |
